# Cholesterol Metabolic Reprogramming in Cancer and Its Pharmacological Modulation as Therapeutic Strategy

**DOI:** 10.3389/fonc.2021.682911

**Published:** 2021-05-24

**Authors:** Isabella Giacomini, Federico Gianfanti, Maria Andrea Desbats, Genny Orso, Massimiliano Berretta, Tommaso Prayer-Galetti, Eugenio Ragazzi, Veronica Cocetta

**Affiliations:** ^1^ Department of Pharmaceutical and Pharmacological Sciences, University of Padova, Padova, Italy; ^2^ Veneto Institute of Molecular Medicine, VIMM, Padova, Italy; ^3^ Department of Clinical and Experimental Medicine, University of Messina, Messina, Italy; ^4^ Department of Surgery, Oncology and Gastroenterology - Urology, University of Padova, Padova, Italy

**Keywords:** cholesterol, cancer, metabolic reprogramming, cancer therapy, pharmacological targeting, pharmacological modulation, metabolic targeting agents

## Abstract

Cholesterol is a ubiquitous sterol with many biological functions, which are crucial for proper cellular signaling and physiology. Indeed, cholesterol is essential in maintaining membrane physical properties, while its metabolism is involved in bile acid production and steroid hormone biosynthesis. Additionally, isoprenoids metabolites of the mevalonate pathway support protein-prenylation and dolichol, ubiquinone and the heme *a* biosynthesis. Cancer cells rely on cholesterol to satisfy their increased nutrient demands and to support their uncontrolled growth, thus promoting tumor development and progression. Indeed, transformed cells reprogram cholesterol metabolism either by increasing its uptake and *de novo* biosynthesis, or deregulating the efflux. Alternatively, tumor can efficiently accumulate cholesterol into lipid droplets and deeply modify the activity of key cholesterol homeostasis regulators. In light of these considerations, altered pathways of cholesterol metabolism might represent intriguing pharmacological targets for the development of exploitable strategies in the context of cancer therapy. Thus, this work aims to discuss the emerging evidence of *in vitro* and *in vivo* studies, as well as clinical trials, on the role of cholesterol pathways in the treatment of cancer, starting from already available cholesterol-lowering drugs (statins or fibrates), and moving towards novel potential pharmacological inhibitors or selective target modulators.

## Introduction

In the last decades, the study of metabolic reprogramming has been revealed as one of the hallmarks of cancer and chemotherapy resistance. It has been demonstrated that cancer cells change their metabolism, increasing glucose demand, glutamine or lipid synthesis, exploiting the pentose phosphate pathway or altering their mitochondrial function, in order to support a higher proliferation rate leading to tumor progression and chemotherapy resistance (1–10). Among these altered pathways, cholesterol metabolic reprogramming has acquired a pivotal role in the field of cancer research.

The discovery of cholesterol dates back to the second half of the eighteenth century, when Poulletier de la Salle isolated for the first time this molecule from human gallstone and bile. Since then, a huge number of researches were undertaken, which eventually led to the understanding of key molecular events of cholesterol biology, such as transport in blood and cellular metabolism. Nowadays, this peculiar lipid is still extensively studied for its involvement in several pathophysiological processes (11, 12). Cholesterol is a ubiquitous sterol found in vertebrate organisms with a plethora of biological functions that are essential for proper cellular growth and activity (13, 14). Due to its alicyclic nature, cholesterol is highly hydrophobic and resides predominantly within the phospholipidic bilayer of cell membranes, where it preserves the barrier function by modulating permeability, fluidity and rigidity (15, 16). In this setting, cholesterol preferentially interacts with the saturated acyl chains of adjacent sphingolipids and glycophosphatidylinositol-anchored proteins of the outer leaflet, forming small ordered and tightly packed microdomains, physically separated from the shorter and unsaturated phospholipids of the bilayer (16–18). These assemblies, usually called lipid rafts, are involved in several biological processes, such as biosynthetic and endocytic vesicular trafficking (19), ceramide-mediated apoptosis (20), host-pathogen interactions (pathogen binding and uptake) (21), cytoskeletal dynamics and rearrangement, cellular polarization (22) and signal transduction (IgE signaling, T-cell antigen receptor signaling, Ras signaling, Hedgehog signaling) (23). Although the most known role of cholesterol as a structural and functional component of cellular membranes is unquestionable (15, 16, 18), it also represents the precursor of bile acids, and its oxidation allows the biosynthesis of steroid hormones in steroid-producing tissues. In addition, the isoprenoid intermediates of the mevalonate pathway can be diverted toward the biosynthesis of dolichol, ubiquinone and the side tail of heme *a* (24, 25), or exploited as substrates for protein-prenylation (26). Lastly, cholesterol has also been found to interact with a large variety of proteins, including receptors, enzymes, etc. by both covalent and non-covalent binding, thus regulating protein stability, localization, and activity. These interactions indicate cholesterol as an important element in the regulation of many biochemical pathways, through the control of protein localization and activity (27).

Due to the crucial role played by this sterol in several physiological settings, disruption of cholesterol homeostasis and metabolic reprogramming may be responsible for the development of cardiovascular disorders and is implicated in the pathogenesis of diabetes, Alzheimer disease and many types of cancer (28–32). Intracellular and systemic cholesterol concentrations are tightly regulated by the balance between *de novo* biosynthesis, uptake, efflux, and storage, and metabolic alterations in lipid/cholesterol pathways have been shown to modulate cancer cells’ sensitivity to chemotherapeutic agents. The dependence of cancer cells on aberrant lipid and cholesterol metabolism could point to these pathways as an attractive target to treat cancer as well as to sensitize them to anticancer therapies (33). Many cholesterol-lowering drugs are approved and used for the treatment of hypercholesterolemia and for the control of pathologies and metabolic disorders. This work focuses on the correlation between cholesterol metabolism and cancer, considering the importance of these pathways in sustaining cell growth, invasion or migration. Furthermore, starting from the relatively recent findings on the role of sterol in tumor progression and chemotherapy response, we will consider how the pharmacological targeting of increased cholesterol metabolism pathways could represent a promising approach for cancer treatment.

## Cholesterol Metabolism

Cholesterol metabolism in humans is complex. Cholesterol is either supplied from the diet (exogenous) or synthesized *de novo* (~70% of total body cholesterol, endogenous). Here below we provide a brief section on the main aspects related to cholesterol metabolism, introductory to understanding the reprogramming aspect observed in cancer cells. For a more accurate description of the fine regulation of cellular processes involving cholesterol, we refer to several specific reviews (34–37).

### Cholesterol Biosynthesis

**Figure 1:**
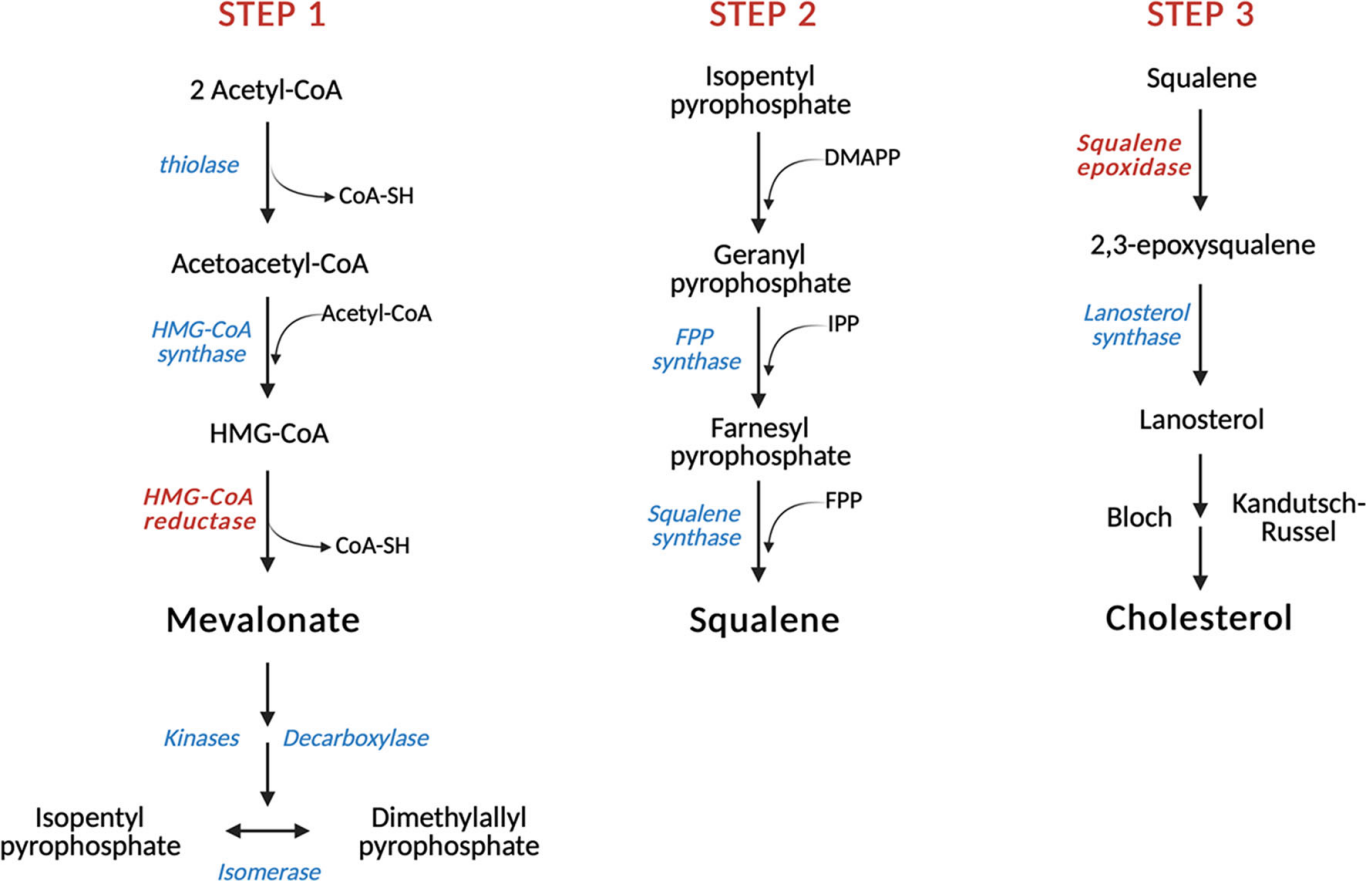
Schematic representation of cholesterol biosynthesis. In the first step of cholesterol biosynthesis, three molecules of acetyl-CoA condense to form HMG-CoA, which is then reduced to mevalonate by the first step-limiting enzyme HMG-CoA reductase (HMGCR). Subsequent reactions allow the conversion of mevalonate into FPP, an isoprenoid that gives rise to squalene in a reaction catalyzed by squalene synthase (SQS). Squalene is then converted by the second rate-limiting enzyme squalene epoxidase (SQLE) into its epoxidic form, which is eventually cyclized to lanosterol by the enzyme lanosterol synthase. Further oxygen-based reactions lead to the formation of cholesterol. Red: rate-limiting enzymes. HMG-CoA, 3-hydroxy-3-methylglutaryl-CoA; IPP, Isopentyl pyrophosphate; DMAPP, Dimethylallyl pyrophosphate; FPP, Farnesyl pyrophosphate.

The biosynthetic cascade which leads to cholesterol production (**Figure 1**) occurs virtually in every mammalian cell, with liver and intestine being the anatomical sites responsible for more than 50% of total cholesterol biosynthesis (38, 39). This process is orchestrated by more than 20 enzymes which are distributed between the cytosol and the endoplasmic reticulum (ER) (40). The first step is catalyzed by the cytoplasmatic enzyme acetylacetyl-CoA thiolase which allows the condensation of two acetyl-CoA molecules to obtain acetylacetyl-CoA. In the second reaction, the enzyme 3-hydroxy-3-methylglutaryl-CoA synthase (HMGCS) allows the introduction of the third molecule of acetyl-CoA for the formation of the branched-chain molecule 3-hydroxy-3-methylglutaryl-CoA, which is then reduced to mevalonate in the first rate-limiting step of cholesterol biosynthesis by 3-hydroxy-3-methylglutaryl-CoA reductase (HMGCR). Afterwards, mevalonate undergoes two subsequent phosphorylations performed by mevalonate kinase (MVK) and phosphomevalonate kinase (PMVK) and an ATP-dependent decarboxylation which eventually yields the isoprenoid precursor isopentyl pyrophosphate (IPP). This intermediate is converted into its isomer dimethylallyl pyrophosphate (DMAPP) in a reversible reaction catalyzed by isopentenyl pyrophosphate isomerase. The condensation of one molecule of IPP with one molecule of DMAPP allows the formation of geranyl pyrophosphate (GPP), which is in turn combined with another IPP molecule by the enzyme farnesyl diphosphate synthase to yield farnesyl pyrophosphate (FPP), a key isoprenoid. At this point, the mevalonate pathway diverts toward the formation of either non-sterol isoprenoids, such as geranylgeranyl pyrophosphate (GGPP), or sterols, through the head-to-head condensation of two FPP molecules, mediated by squalene synthase, which gives rise to squalene. Intracellular accumulation of non-sterol products is required for post-translational modification processes (N-glycosylation and Cys-prenylation) of diverse proteins that play important roles in cellular growth and signal transduction (24, 41). On the other hand, squalene epoxidase (SQLE), the other rate-limiting enzyme of cholesterol biosynthesis, converts squalene into its epoxydic form 2,3-epoxysqualene, which is then cyclized to lanosterol by the enzyme lanosterol synthase. The last phase of cholesterol biosynthesis involves 19 oxygen-based reactions which include demethylations, double-bond reductions, and double bond replacements. In this context, lanosterol enters the Bloch branch or the Kandutsch–Russell pathway and is processed through the formation of several intermediates which yields desmosterol and 7-dehydrocholesterol, the direct precursors of cholesterol (42–44). Recently, the existence of a third hybrid pathway has also been suggested for the conversion of lanosterol into cholesterol (45).

### Cholesterol Uptake and Efflux

The dietary intake of cholesterol is extremely important to ensure the maintenance of its homeostasis (46, 47). In the small intestinal lumen, dietary sterols are solubilized into micelles by bile acids and adsorbed in a process facilitated by the Niemann–Pick C1-like-1 (NPC1L1) protein, which is localized in the apical membrane of enterocytes and allows cholesterol uptake in a clathrin-mediated endocytosis fashion (48, 49). Once inside the enterocyte, cholesterol is mainly converted to cholesteryl esters by the ER enzyme acyl-coenzymeA cholesterol acetyltransferase 2 (ACAT2) and then packed into nascent chylomicrons, together with dietary triglycerides and apolipoprotein B-48 (50, 51). Through the lymphatic system, chylomicrons are poured into the bloodstream and metabolized by the endothelial enzyme lipoprotein lipase, which hydrolyzes the triglycerides contained in the core to yield chylomicron remnants. The released fatty acids are used by peripheral tissues including muscles and adipose tissue either for storage or oxidation, while dietary cholesterol is delivered to the liver by chylomicron remnants (52, 53). Hepatic cholesterol and triglycerides are coupled to apolipoprotein B and incorporated into VLDL particles, which are secreted into the blood and hydrolyzed by plasma lipases to yield IDL. IDLs are further converted into LDLs, particles rich in cholesterol and cholesteryl esters which are captured by LDL receptor-expressing tissues, including the liver and other extrahepatic tissues (54, 55). On the other hand, LDL is driven towards the lysosomal compartment where lysosomal lipases hydrolyze the cholesteryl esters stored in the core to cholesterol, which eventually exits from the lysosome lumen aided by the coordinated action of NPC1, NPC2 and LAMP2 and reaches other cellular organelles, mostly *via* non-vesicular transport mediated by sterol transfer proteins (STPs) (56–58). Cholesterol elimination also significantly impacts cellular homeostasis. Therefore, the excess of cellular cholesterol in peripheral tissues has to be stored as less-toxic cholesteryl esters in lipid droplets or disposed and moved towards the liver for recycling or excretion, by a process usually referred to as reverse cholesterol transport (59, 60). Cholesterol removal from extrahepatic cells is driven by HDL particles, which accumulate and transport cholesteryl esters to the liver, the adrenal glands, and the gonads (61). Cholesteryl esters are then converted to free cholesterol by cholesteryl ester hydrolase (CEH) for either steroid hormones synthesis in steroid-producing organs or cholesterol excretion and bile acids synthesis in the liver (62, 63). Cellular cholesterol efflux is controlled by four regulatory proteins belonging to the ATP-binding cassette (ABC) transporter superfamily, namely ABCA1, ABCG1, ABCG5 and ABCG8. ABCA1 mediates the transport of cholesterol and phospholipids to lipid-free apolipoprotein A-I (apo A-I) in the blood allowing the generation of nascent discoidal HDL particles, which are converted into globular and mature HDLs under the action of lecithin:cholesterol acyl transferase (LCAT) by accepting further cholesterol from ABCG1 (63, 64).

### Cholesterol Storage

Intracellular cholesterol excess is usually esterified by the ER enzyme acyl coenzyme A cholesterol acetyltransferase (ACAT), which catalyzes the transfer of a fatty acyl group to cholesterol (65). Indeed, ACAT-produced cholesteryl esters can be easily stored into lipid droplets preventing free-cholesterol lipotoxicity (66). Cholesterol esterification is also involved in lipoprotein and steroid hormone production, as well as in chylomicrons assembly for cholesterol absorption (67). Two ACAT isoenzymes have been identified in mammals so far, consistent with their different tissue distribution. ACAT1 is widely expressed throughout the body, suggesting its involvement in maintaining cholesterol homeostasis, while ACAT2 expression is exclusive in enterocytes and hepatocytes, where it contributes to lipoprotein biosynthesis and assembly (68, 69).

### Regulation of Cholesterol Homeostasis

In order to ensure the maintenance of cellular and systemic cholesterol homeostasis, mammalian cells must carefully orchestrate the set of molecular pathways involved in cholesterol biosynthesis, uptake, storage and efflux (70, 71). This is accomplished by sterol-sensitive systems, which couple variations in cellular sterol levels with adaptive responses. Particularly, three adaptive factors are considered as key regulators of cholesterol homeostasis, namely sterol regulatory element-binding protein-2 (SREBP2), liver X receptors (LXRs) and nuclear factor erythroid 2 related factor-1 (NRF1) (40). SREBP2 belongs to the basic-helix-loop-helix-leucine zipper (bHLH-Zip) family of transcription factors and lies within the ER membrane associated with SREBP-cleavage activating protein (SCAP) through its C-terminal portion (72). The N-terminal transcription factor portion, usually referred to as nuclear SREBP2 (nSREBP2), undergoes dimerization and is then imported inside the nucleus, where binds to sterol responsive elements (SREs) in the promoter regions of target genes, inducing their transcription (73, 74). Conversely, when ER-membrane cholesterol levels increase above the threshold, the sterol sensitive domain (SSD) of SCAP binds to cholesterol and SCAP switches to an open conformation promoting its interaction with insulin-induced gene 1 (INSIG1) protein (75). nSREBP2 binds to and induces the transcription of HMGCR and SQLE genes, which encode for the two rate-limiting enzymes of cholesterol biosynthesis, increasing sterols intracellular levels (76, 77). HMGCR levels are also regulated either by direct interaction with ER-sterols through its SSD (INSIG1-mediated ubiquitination) or by covalent modification (AMPK-mediated phosphorylation) (78, 79). SREBP2 activation also increases the expression of NPC1L1 and LDLR genes, two master regulators of cholesterol intestinal absorption and cholesterol intake by peripheral cells, respectively (80, 81). Moreover, a SRE motif is contained upstream of SREBP2 gene, suggesting that nSREBP2 promotes the activation of its own gene (feed-forward mechanism) (82). Under increasing cholesterol levels, the ER preserves cellular homeostasis by recruiting the adaptive factor NRF1 (61). NRF1 resides within the ER-membrane but is rapidly activated by proteolysis, released from the ER and translocated into the nucleus, where it regulates the transcription of its target genes by binding to anti-oxidant response elements (AREs) (83). Particularly, when NRF1 is activated and enters the nucleus, it represses the transcriptional activity of LXR, which promotes cholesterol excretion, export and storage, while inhibiting *de novo* biosynthesis. Differentially to SREBP2 and NRF1, LXRs are nuclear receptors which, upon heterodimerization with the retinoid X receptor-α (RXR α), bind to LXR responsive elements (LXRE) and regulate the expression of several genes involved in lipid homeostasis (84). Once activated, LXRs promotes the activation of genes involved in bile acids production (CYP7A1), cholesterol excretion (ABCG5, ABCG8) and reverse cholesterol transport (ABCA1, ABCG1) (85–87). Moreover, LXRs impair cholesterol intestinal absorption by down-regulating NPC1L1 expression and inhibit cholesterol cellular uptake by promoting IDOL-mediated LDLR degradation (88–90). Overall, LXRs activity prevents lipotoxicity induced by intracellular accumulation of sterols.

### Cholesterol Lowering Drugs

Since cholesterol plays a key role in many cellular processes, disruption of cholesterol homeostasis is linked to the onset of several diseases, including metabolic disorders, atherosclerosis, cancer, etc. Several therapeutic classes of drugs are currently used to treat hypercholesterolemia (**Table 1**) and to prevent associated cardiovascular diseases (110). Statins are the first-line treatment of hypercholesterolemia and they have an important role in the prevention of cardiovascular diseases. Statins are competitive inhibitors of 3-hydroxy-3-methylglutaryl-CoA reductase (HMGCR), the enzyme responsible for the reduction of HMG-CoA into mevalonate (91). This specific block causes effects on cholesterol metabolism, such as diminished plasma triglycerides, enhanced HDL, and upregulation of LDL receptor (LDLR) expression, which leads to increased LDL uptake in hepatocytes and decreased blood LDL content (92). Fibrates are another therapeutic class of drugs prescribed to treat hypercholesterolemia (98, 99). They are agonists of the transcription factor PPARα, that once activated, translocates in the nucleus, heterodimerizes with the retinoid X receptor (RXR) and binds to peroxisome proliferator response elements (PPREs) starting the gene’s transcription (100). The effects on lipoprotein metabolism and cellular cholesterol homeostasis are decreased hepatic synthesis and decreased serum levels of triglycerides, reduced synthesis of VLDL, increased HDL cholesterol, and regulation in fatty acid synthesis and uptake, such as regulation of FAT or CD-36 (98, 99). Other therapeutic classes of cholesterol-lowering drugs are represented by selective cholesterol absorption inhibitors, such as ezetimibe; resins, such as cholestyramine, colestipol and colesevelam, which are bile acid sequestrants (103); apolipoprotein B synthesis inhibitors, such as mipomersen (105); microsomal transfer protein inhibitors, such as lomitapide (105). A new promising therapeutic class of cholesterol-lowering drugs is represented by PCSK9 inhibitors. PCSK9 is predominantly produced in hepatocytes, where it decreases LDLR number. When PCSK9 binds LDLR there is a consequent block of LDLR in an open conformation and its recycling is blocked. Then, LDLR is degraded by lysosomes (109). Another recently approved cholesterol-lowering drug is bempedoic acid (8-hydroxy-2,2,14,14-tetramethylpentadecanedioic acid) (97), acting as ATP citrate lyase inhibitor, an enzyme upstream from 3-hydroxy-3-methylglutaryl-CoA. Since the focus of this review is the repositioning of cholesterol-lowering drugs in oncology, we refer to **Table 1** for a schematic explanation of the mechanisms of action, effects on cholesterol metabolism, and possible side effects of the drugs.

**Table 1:** Current cholesterol-lowering drugs and relative mechanism of action, main effects on cholesterol metabolism, adverse effects and therapeutic indications.

Therapeutic class	Drug	Mechanism of action	Effects on cholesterol metabolism	Main adverse effects	Clinical indications	References
**Statins**	Lovastatin	Competitive inhibitors of HMGCR		Myalgia, myositis, rhabdomyolysis	Primary H, Mixed dyslipidemia	(91–96)
	Simvastatin		↑HDL			
	Pravastatin		↓plasma triglycerides			
	Fluvastatin		↑*LDLR*			
	Rosuvastatin		↓LDL			
	Atorvastatin					
	Pitavastatin					
**Bempedoic acid**	Bempedoic acid (prodrug)	Inhibitor of ATP citrate lyase	↓LDL	Myalgia, muscular disorders, gout	Primary H, Mixed dyslipidemia	(97)
**Fibrates**	Gemfibrozil	*PPARα* agonists	↑HDL	Nausea, abdominal pain	Primary H, Mixed dyslipidemia	(98–100)
	Fenofibrate		↓serum triglycerides			
	Fenofibric acid		↓VLDL			
**Selective cholesterol absorption inhibitors**	Ezetimibe	Selective block of NPC1L1	↑*LDLR*	Myopathy, acute pancreatitis (when it is combined with statins)	Dyslipidemia, FH	(101, 102)
			↓serum LDL			
			↑HDL			
			↓triglycerides			
**Resins**	Cholesthyramine	Bile acid binders	↑HDL	Gastrointestinal effects	Dyslipidemia, Primary H, H associated with mild HT	(103, 104)
	Colesevelam		↓LDL			
	Colestipol		↑serum triglycerides			
**Apolipoprotein B synthesis inhibitor**	Mipomersen	Second-generation antisense oligonucleotide inhibitor of apoB-100	↓apoB	Injections site reactions; flu-like symptoms; elevated transaminasis (alanine aminotransferase) → reversible; hepatic steatosis → reversible	FH, Severe H	(105–107)
			↓LDL			
			↓VLDL			
			↓lipoprotein			
**Microsomal transfer protein inhibitor**	Lomitapide	Inhibitor of the microsomal triglyceride transfer protein (MTTP)	↓VLDL	Diarrhoea, nausea, dyspepsia, vomiting;	Adult	(108)
			↓LDL	elevated liver aminotransferase	HoFH	
**PCSK9 inhibitors**	Alirocumab	Inhibition of PCSK9	↓*LDLR*	Nasopharyngitis	FH	(109)
	Evolocumab		↓LDL			

HMGCR, 3-hydroxy-3-methyl-glutaryl-coenzyme A reductase; PPARα, Peroxisome proliferator-activated receptor alpha; LDLR, low density lipoprotein receptor; HDL, High Density Lipoproteins; LDL, Low Density Lipoproteins; VLDL, Very Low Density Lipoproteins; MTTP, microsomal triglyceride transfer protein; HoFH, Homozygous familial hypercholesterolaemia; FH, familial hypercholesterolaemia; H, hypercholesterolaemia; PCSK9, proprotein convertase subtilisin/kexin type 9; HT, hypertriglyceridemia.

## Cholesterol Metabolic Reprogramming in Cancer: Pharmacological Targeting

Cancer cells are highly proliferative and therefore strongly dependent on cholesterol to satisfy their increasing demand of substrates for membrane biosynthesis (111). Accordingly, cholesterol is generally beneficial for cancer growth and development, as it promotes oncogenic signaling and evasion of apoptosis, as well as cell migration and invasion (112–115). Notably, cancer cells increase their cholesterol demand by enhancing *de novo* biosynthesis or uptake, by altering the cholesterol efflux, or by increasing its storage, as will be described in the following sections. Also, cholesterol homeostasis is largely compromised in cancer development and progression, as will be discussed below. In line with this, the differential cholesterol requirements of tumors provide novel therapeutic strategies for the treatment of several malignancies. As above mentioned, the purpose of this review is to sum up the main alterations in cholesterol-related metabolic pathways observed in *in vitro* and *in vivo* cancer models. The current scientific evidence highlights the reprogramming of lipid/cholesterol pathways in many cancers, thus suggesting intriguing targets exploitable for a combined therapy with conventional chemotherapeutic agents in the fight against cancer. In the following sections we will describe the main alterations observed in cancer cells in the context of cholesterol metabolism, pointing out the more interesting targets identified since now. The identification of specific targets has opened the possibility to exploit them for a pharmacological approach by using cholesterol lowering/modulating drugs. Here we will review the current literature focused on the use of cholesterol targeting drugs in the context of cancer treatment. Results from *in vitro* and *in vivo* studies have allowed the translation into clinical trials of some drugs that are discussed in the following sections, highlighting the potential of this combined approach for cancer treatment (**Table 2**).

**Table 2:** Sum up of drugs acting on cholesterol metabolism investigated as potential treatment in cancer therapy.

Target	Drug	Cancer	Preclinical/clinical phase	References
OSC	**Ro 48-8071**	Breast cancer	*In vitro* and *in vivo* studies	(116, 117)
		Colon carcinoma	*In vivo* studies	(118)
		Pancreatic ductal denocarcinoma	*In vivo* studies	(118)
		Hormone-dependent and castration-resistant prostate cancer	*In vitro* and *in vivo* studies	(119)
SQS	**Zaragozic acid**	Prostate cancer	*In vitro* studies	(120)
		RMA lymphoma	*In vivo* studies	(121)
		Lewis lung carcinoma	*In vivo* studies	(121)
ACAT-1	**CP-113818**	Breast cancer	*In vitro* studies	(122)
	**Bitter melon extract**	Breast cancer	*In vivo* studies	(123)
	**Avasimin (nanoformulation with avasimibe)**	Prostate, pancreatic, colon and lung cancers	*In vitro* and *in vivo* studies	(124)
		Metastatic prostate cancer	*In vivo* studies	(125)
		Pancreatic cancer	*In vitro* and *in vivo* studies	(126)
	**Avasimibe**	Metastatic prostate cancer	*In vitro* studies	(125)
		Pancreatic ductal adenocarcinoma	*In vitro* studies	(126)
		Lewis lung cancer	*In vitro* and *in vivo* studies	(127)
	**Avasimibe + gemcitabine**	Pancreatic ductal adenocarcinoma	*In vitro* and *in vivo* studies	(128)
	**Avasimibe + cyclophosphamide**	Lewis lung cancer	*In vivo* studies	(127)
LXR	**T0901317**	Prostate cancer	*In vitro* and *in vivo* studies	(129)
		Breast cancer	*In vitro* studies	(130)
		Melanoma	*In vitro* and *in vivo* studies	(131)
		Multiple myeloma	*In vitro* and *in vivo* studies	(132)
		Oral squamous cell carcinoma	*In vitro* and *in vivo* studies	(133)
		Ovarian cancer	*In vitro* studies	(134)
	**T0901317 + Gefitinib**	Lung cancer	*In vitro* and *in vivo* studies	(135)
	**T0901317 + Sorafenib**	Hepatocellular carcinoma	*In vitro* and *in vivo* studies	(136)
	**22(*R*)-hydroxycholesterol**	Breast cancer	*In vitro* studies	(130)
		Multiple myeloma	*In vitro* studies	(132)
	**GW3965**	Breast cancer	*In vitro* studies	(137, 138)
		Melanoma	*In vivo* studies	(139)
		Pancreatic ductal adenocarcinoma	*In vitro* studies	(140)
		Multiple myeloma	*In vitro* studies	(132)
		Colon cancers	*In vitro* studies	(138)
	**GW3965 + Gefitinib**	Lung cancer	*In vitro* studies	(141)
	**SR9243**	Prostate, lung, colon cancers and clear cell renal cell carcinoma	*In vitro* and *in vivo* studies	(142, 143)
	**LXR623**	Clear cell renal cell carcinoma	*In vitro* studies	(143)
PPARα	**Fenofibrate**	Ishikawa endometrial cancer	*In vitro* studies	(144)
		Hepatoma	*In vitro* studies	(145)
		Oral cancer	*In vitro* and *in vivo* studies	(146)
		Gastric cancer	*In vitro* and *in vivo* studies	(147)
		Low-grade glioma and ependymoma	Phase-II clinical trial	(148)
	**Fenofibrate + retinoic acid**	Ishikawa endometrial cancer	*In vitro* studies	(144)
	**Fenofibrate + docetaxel**	Prostate cancer	*In vitro* studies	(149)
SREBP	**Fatostatin**	Prostate cancer	*In vitro* and *in vivo* studies	(150)
		Endometrial cancer	*In vitro* and *in vivo* studies	(151, 152)
		Breast cancer	*In vitro* and *in vivo* studies	(153)
	**Fatostatin + Tamoxifen**	Breast cancer	*In vitro* studies	(154)

### Enhanced Cholesterol *de novo* Biosynthesis

**Figure 2:**
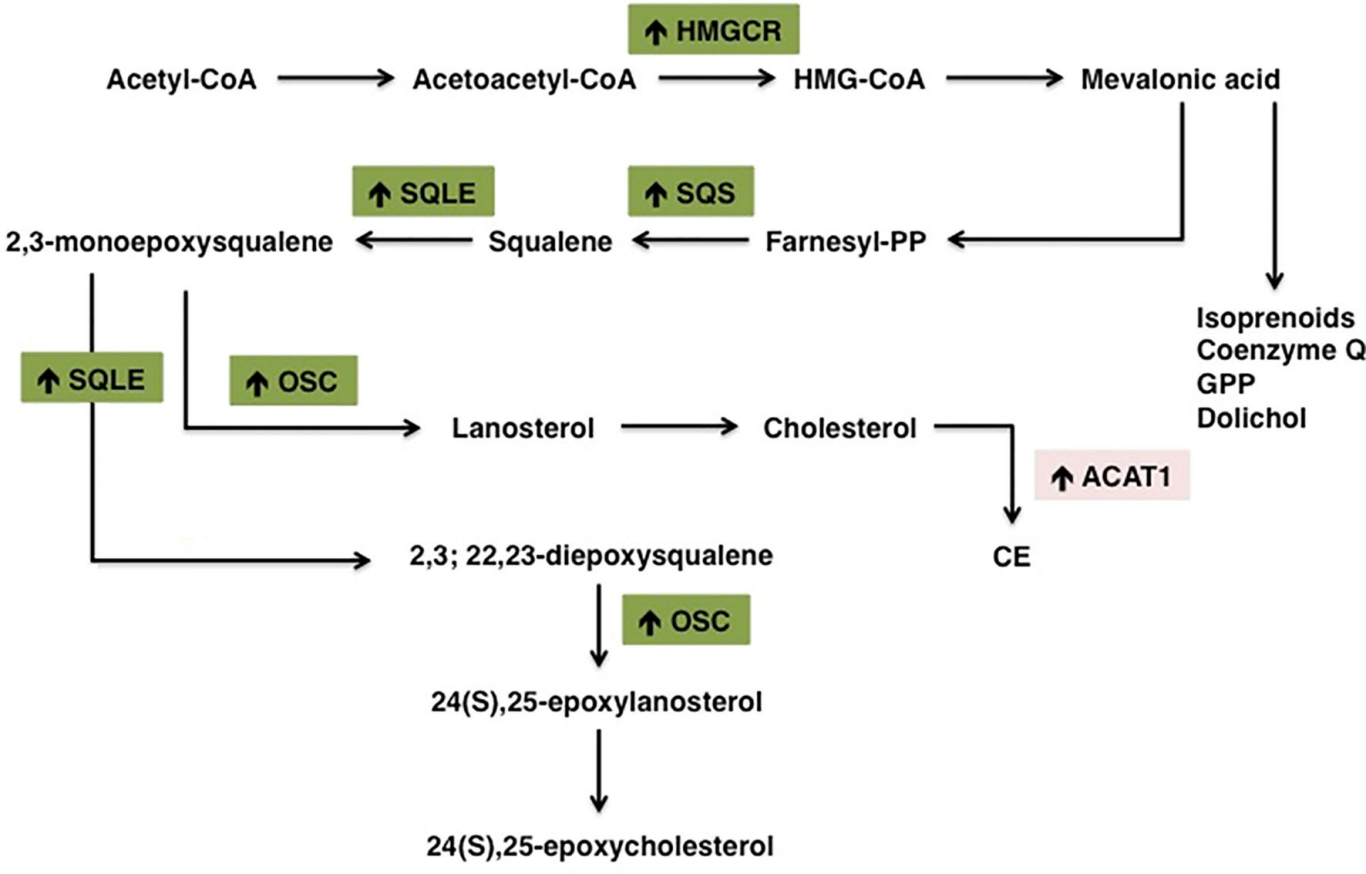
Schematic representation of the main alterations in cholesterol metabolism pathway in tumors. Cancer cells are highly proliferative and therefore strongly dependent on cholesterol to sustain the high demand of substrates for membrane biosynthesis. Cancer cells increase their cholesterol demand by enhancing *de novo* biosynthesis (or exogenous uptake). Increased/overexpressed enzymes in cholesterol biosynthesis pathway are indicated with (↑). HMGCR, 3-hydroxy-3-methylglutaryl-CoA reductase; SQS, Squalene synthase; SQLE, Squalene epoxidase; ACAT1, Acetyl-CoA Acetyltransferase 1.

Many cancers upregulate *de novo* cholesterol biosynthesis, thereby fueling the oncogenic machinery and sustaining tumor progression (155). Aberrant cholesterol biosynthetic program can be considered as a hallmark of transformed cancer cells and has been correlated with lower overall patient survival in melanoma, acute myeloid leukemia and sarcoma (32). Consistently, in breast cancer cholesterol biosynthesis-related genes are considered reliable prognostic factors associated with shorter relapse-free survival (156). Cholesterol biogenesis is carried on through the mevalonate pathway (**Figure 2**), which leads to the production of farnesyl pyrophosphate (FPP), responsible for the formation of either the non-sterol isoprenoid geranylgeranyl pyrophosphate (GGPP) or squalene. The first rate-limiting enzyme HMGCR is overexpressed in many tumors, such as prostate cancer, gastric cancer and colon cancer (157–159). Indeed, the accumulation of non-sterol isoprenoids mediates several oncogenic activities by post-translationally modifying key proteins directly involved in the expression of oncogenes, cytoskeletal organization and cell survival/proliferation (24, 160). This process is collectively known as protein prenylation and allows the covalent attachment of lipid moieties to small oncogenic G proteins, thereby promoting their activation and transforming function (26). GTP-binding proteins Rho, Rac, Rab, Rap, Ras (Ras, Rho, Rab superfamily of GTPases) are all dependent from farnesylation and geranylgeranylation to exert their tumorigenic activities, which eventually promote cell cycle progression and cellular survival, as well as tumor cells motility, migration and metastasis (41, 161, 162). Moreover, isoprenoids are involved in ubiquinone biogenesis. Ubiquinone (CoQ) is a redox active lipid that functions as electron carrier in the mitochondrial respiratory chain. CoQ sustains p53-deficient colon cancer cells growth and development by promoting *de novo* pyrimidine synthesis and maintaining the integrity of the electron transfer chain even under nutrients starvation and oxygen restriction (163). Differently from steroidogenic healthy tissues, HMGCR activity in tumors is refractory to sterol-mediated negative feedback regulation (36, 164). Therefore, HMGCR altered regulation allows the accumulation of isoprenoids even in cholesterol-enriched conditions, thereby sustaining the production of non-sterol mevalonate intermediates essential for the establishment of tumor malignant phenotype (41). The alternative branch of mevalonate pathway diverts towards the formation of sterols through the activity of squalene synthase (SQS), which gives rise to squalene. In lung cancer patients, SQS is frequently overexpressed and associated with poor prognosis and tumor metastasis. Indeed, the enhanced expression of SQS induces cholesterol biosynthesis, which in turn sustains Tumor Necrosis Factor Receptor 1 (TNFR1) accumulation into lipid rafts and subsequent NF-κB and MMP1 activation (165). Squalene epoxidase (SQLE) converts squalene into squalene-2,3-epoxide and represents the other rate-limiting enzyme in sterol biogenesis. SQLE activity is dysregulated in many tumors, such as breast, lung and colorectal cancer (166–168). Colorectal tumors are characterized by higher SQLE expression levels when compared with healthy tissues, which sustain tumor development by promoting extracellular signal-regulated kinase 1/2 (ERK1/2) oncogenic activity (169). Similarly, in breast cancer SQLE is frequently amplified at the gene level and strongly overexpressed in more aggressive and undifferentiated tumors, thereby demonstrating its oncogenic potential (170). On the other hand, a subset of tumors presents SQLE downregulation and subsequent cholesterol auxotrophy. Lymphoma SQLE-deficient cells accumulate squalene, which modifies cellular membranes and lipid droplets composition, thereby protecting neoplastic cells from the oxidative damage and ferroptosis (171, 172). In breast cancer cells, NAD(P)H-dependent steroid dehydrogenase-like protein (NSDHL) and sterol-C4-methyl oxidase (SC4MOL), two enzymes of the Kandutsch-Russell pathway, are overexpressed and translocate to the plasma membrane. Here, they promote metastasis development by modulating lipid rafts’ sterol composition (173, 174). Another key post-squalenic enzyme is oxidosqualene cyclase (OSC), which mediates 2,3-oxidosqualene cyclization into lanosterol (175). In metastatic mouse models of human colorectal and pancreatic cancer, OSC promotes tumor neovascularization and metastatic potential. Consistently, OSC inhibitors hamper endothelial cell migration and promote cell apoptosis, thereby inhibiting tumor-associated angiogenesis and dissemination to distal organs (116, 118). Additionally, OSC plays an important role in cell self-renewal and its expression is increased in breast cancer stem cells (156, 176). In conclusion, the mevalonate pathway is oncogenic at many levels and frequently dysregulated in several cancers.

#### Pharmacological Modulation of HMGCR

As mentioned, HMGCR is the rate-limiting enzyme of the mevalonate pathway, which produces cholesterol. Considering the overexpression of this enzyme in many tumors, targeting HMGCR could be a good strategy for cancer clinical therapy. As stated, statins are the commonest pharmacological inhibitors of HMGCR and the repositioning of these drugs in the cancer field is well studied and established. Statins exerts antitumor activities with different molecular mechanisms, such as reducing cell proliferation or tumor cell survival (177), suppressing angiogenesis or causing apoptosis, and reducing tumor growth and metastasis (178–180). The efficacy of these drugs has been evaluated both in monotherapy and in combination therapy with standard chemotherapeutic agents. *In vitro* studies evidenced that statins are able to inhibit cell proliferation and viability, or causing apoptosis in different human cancer cell lines, such as breast, ovarian and prostate tumor cells. In particular, it has been demonstrated that the open-ring conformation of statins is responsible for the inhibition of HMGCR and apoptosis induction (181). *In vivo* studies evidenced that statins reduce tumor growth and arrest metastasis progression (113, 182–186). Terzi and co-workers (187) evaluated the effect of two statins, atorvastatin and simvastatin, combined with the standard chemotherapeutic agent, bortezomib, in human multiple myeloma. The results showed that statins are able to improve the effectiveness of bortezomib and reduce the adverse effects (187). The combined treatment of pivastatin with gemcitabine synergically reduced cell proliferation of MIA PaCa-2 cells inducing cell cycle arrest. Moreover, the same combination reduced the tumor growth in *in vivo* xenograft models (188). A new formulation of atorvastatin was developed in order to cross the blood-brain barrier and target the glioblastoma tumor cells. This new nanoformulation was cytotoxic in mouse and human glioblastoma cells, and was able to reduce the growth in a three-dimensional (3D) tumor model (189). The anticancer role of statin treatment in combination with conventional anticancer drugs, has been tested in clinical trials for the treatment of different types of tumors, such as breast, prostate, ovarian or lung cancers, bringing to light controversial effects of the association (**Table 3** summarizes some clinical trials on breast, ovarian and prostate cancer). Different clinical trials demonstrated that statins are able to reduce tumor progression and enhance the survival rate of patients with breast cancer (194). Farooqui reviewed different randomized controlled trials and concluded that the addition of statins to standard chemotherapy is not able to enhance the survival in patients with advanced cancers and a prognosis of less than 2 years (195). A Swedish study, instead, concluded that statins use for 6 months in patients with multiple myeloma enhanced the survival rate of both men and women (196). In another study, atorvastatin was administered in patients with prostate cancer for 27 days before prostatectomy. In this case, drug administration was not able to decrease tumor proliferation with respect to placebo, however, a longer administration of atorvastatin showed beneficial effects (197). In light of this result, it could be interesting to define the specific chronic drug administration before surgery. In fact, other clinical trials evidenced that a chronic therapy of 6 months before surgery resulted to be more efficient compared with a 3 months therapy in reducing tumors (198). It is thus evident that the current knowledge obtained from several *in vitro* and *in vivo* studies in different types of tumors confirmed and deepened the molecular mechanisms of statins as anticancer drugs. Since statins were already approved for hypercholesterolemia treatment, their repositioning in the oncological field has benefited from an easier and faster translation into clinic. However, clinical trials evaluating the antitumor effect of these drugs are still few. Monotherapy studies highlight the potential of this class of drugs in cancer prevention, but the pharmacological differences among all statins, as well as the heterogeneity of tested tumors, lead to a lack of proven positive outcomes. In addition, it has been demonstrated the impossibility to administer high doses because of their adverse effects (199). At the same time, relatively few clinical trials take into consideration the combined therapy with standard chemotherapeutic agents, a strategy allowing low dose administration and lower toxicity of the single agents. Thus, further studies evaluating the beneficial effect of statins in combination with conventional chemotherapy have to be conducted in order to assess their potential in cancer therapy.

**Table 3:** Statins currently under evaluation in clinical trials for cancer treatment.

Type of statin	Cancer	Aim	Phase trial	ClinicalTrials.gov Identifier
**Simvastatin**	Breast cancer	Identify the molecular and genetic mechanisms by which statins influence breast cancer cell proliferation	Recruiting – Phase II	NCT03454529
		Preventive effect of a new breast cancer for women with high risk of a new breast cancer	Completed – phase II	NCT00334542 (190)
		Combined therapy with anti-HER2 to sensitize it in metastatic cancer	Recruiting- Phase II	NCT03324425
	Gastric cancer	Combined therapy with Capecitabine/cisplatin did not increase the progression-free survival of patients with advanced cancer	Completed- Phase III	NCT01099085 (191)
	Ovarian cancer	To evaluate the effect in women with ovarian cancer platinum sensitive	Recruiting – early phase I	NCT04457089
**Lovastatin**	Breast cancer	Chemoprevention effect of statin in women with high cancer risk	Completed – Phase II trial	NCT00285857 (192)
	Ovarian cancer	Combined therapy of paclitaxel and lovastatin in refractory or relapsed ovarian cancer	Completed – Phase II trial	NCT00585052
**Rosuvastatin**	Non-small cell lung cancer	Combined therapy with erlotinib in advanced incurable cancer	Completed –Phase I trial	NCT00966472
	Colorectal cancer	To study the effect in patients with stage I or II cancer after surgery	Terminated – Phase III	NCT01011478
	Rectal cancer	Combined with standard chemoradiation to enhance the patients’ survival	Recruiting- phase II	NCT02569645
**Fluvastatin**	Breast cancer	Effect of statin on biomarkers in women who are undergoing surgery	Completed – Phase II trial	NCT00416403 (193)
**Atorvastatin**	Triple negative breast cancer	Antitumor effect of combined treatment of atorvastatin and zoledronate	Recruiting – Phase II trial	NCT03358017
	Prostate cancer	Effect of its administration before prostatectomy	Completed-Phase II trial	NCT01821404

#### Pharmacological Modulation of OSC

2,3-Oxidosqualene cyclase (OSC) is the enzyme that catalyzes the conversion of 2, 3-monoepoxysqualene into lanosterol acting downstream of HMGCR (116). Since lanosterol is the precursor of cholesterol, the inhibition of OSC causes decreased cholesterol synthesis (175), but unlike statins the LDL catabolism is not affected. This suggests that OSC inhibitors act in a different way (200, 201). Staedler et al. (175) demonstrated the antitumor effect of OSC inhibitors in human glioblastoma and brain-derived endothelial cells. Moreover, the combined treatment with OSC inhibitors and atorvastatin showed an increased antitumor effect in human glioblastoma cells with respect to monotherapy (175). Among the OSC inhibitors, Ro 48-8071 ([4^’^ -[6-(Allylmethylamino)hexyloxy]-4-bromo-2^’^ - fluorobenzophenone fumarate]) emerged for its potential antitumor effect. Grinter and coworkers (117) demonstrated that this molecule was able to inhibit cell proliferation in BT-474 human breast cancer cells (117). Ro 48-8071 decreased cell viability of ERα-positive human breast cancer cells (BT-474 and MCF-7), without affecting normal AG11132A cells. Moreover, this compound proved efficacy in reducing tumor growth in *in vivo* mouse xenograft model injected with BT-474 cells, without showing toxicity at doses administered (116). Maione et al. (118) demonstrated the antitumor effect of Ro 48-8071 in murine models of intestine and pancreas cancers. In fact, the role of this compound was investigated in mouse xenograft models injected with human colon carcinoma HCT116 cells, and pancreatic ductal adenocarcinoma HPAF-II cells. Results evidenced that Ro 48-8071 was able to weaken angiogenesis and inhibit tumor growth in the two mouse models previously mentioned (118). Hyder and coworkers (119) investigated the effect of Ro 48-8071 on cell viability and apoptosis in different lines of human prostate cancer cells. Data showed that this compound reduced cell viability in hormone-dependent LNCaP, castration-resistant PC-3 and DU145 prostate cancer cells, also causing apoptosis (119). Since several studies demonstrated the antiproliferative role of ER-β (202–205), castration-resistant prostate cancer cells were treated with the combination of Ro 48-8071 and ER-β agonist diarylpropionitrile showing enhanced activity in inhibiting cell viability. *In vivo* treatment with Ro 48-8071 was able to suppress the growth of prostate cancer PC-3 cell xenografts in mice (119).

#### Pharmacological Modulation of SQS

Squalene synthase (SQS) is an enzyme that catalyzes the conversion of FPP into squalene, the precursor of cholesterol (206). Since squalene synthase is the enzyme responsible for the first committed step in cholesterol production, its targeting results of interest in clinical therapy (207). Zaragozic acids, natural products obtained from fungi, are pharmacological inhibitors of SQS. Brusselmans et al. (120) demonstrated that the expression of squalene synthase was enhanced in LNCaP prostate cancer cells following androgen stimulation. Thus, the inhibition of the enzyme by downregulation or by treatment with zaragozic acid A, was able to cause both the arrest in growth and the induction of cytotoxicity in prostate cancer cells (120). Lanterna and coworkers (121) tested two different isoforms of zaragozic acid, A and B, in mouse models of RMA lymphoma and LLC Lewis lung carcinoma and results confirmed the ability of zaragozic acid in reducing tumor growth, without showing adverse effects (121).

### Enhanced Cholesterol Uptake

An alternative strategy exploited by cancer cells to promote sterol-mediated proliferation is to increase the uptake of exogenous cholesterol. NPC1L1 is a critical sterol transporter, essential for cholesterol intestinal uptake. In colorectal cancer, NPC1L1 promotes colitis-associated tumorigenesis by inducing cholesterol absorption and increasing its plasmatic levels (208). Malignant cells capture and internalize cholesterol through the activity of LDLRs. Indeed, LDLRs expression levels are increased in many cancers, including glioblastoma and leukemia, as well as in pancreatic and lung cancers (209). Also, higher levels of LDLRs negatively correlate with the survival of patients affected by pancreatic adenocarcinoma (210). HER2-positive and triple-negative breast tumors characterized by higher LDLRs intratumoral levels are associated with poorer prognosis, suggesting an important contribution of LDL cholesterol in breast cancer progression (211). LDLRs promote tumor development and progression, by modulating cancer cell invasive and migratory potential, as well as adhesivity and plasticity. Indeed, LDLRs foster epithelial-to-mesenchymal transition (EMT), the secretion of metalloproteinase MMP-9 and the activation of Wnt/β-catenin oncogenic signaling (212, 213). On the other hand, HDL cholesterol is accumulated by steroidogenic organs in a process mediated by scavenger receptor type B class 1 (SRB1). SRB1 is overexpressed in diverse human malignancies, such as prostate, breast, ovarian, and colorectal cancers (214). In lung adenocarcinoma, SRB1 represents an independent prognostic factor and its expression positively correlates with malignant tumor behavior and impaired overall survival (215). Consistently, cancer patients present lower levels of HDL cholesterol compared to healthy subjects, suggesting that cancer cells exploit HDL cholesterol from peripheral tissues to sustain their malignant phenotype by picking it up in a SRB1-mediated fashion (36). Indeed, high levels of SRB1 characterize highly undifferentiated and metastatic prostate tumors, which are usually associated with androgen independence. Accordingly, SRB1-mediated cholesterol supplying might provide sterol precursors, thereby promoting tumor self-production of androgens and the development of castration-resistant phenotypes (216). Currently, there are not pharmacological inhibitors targeting LDLR and SRB1. However, increasing evidence highlighted the correlation between high-cholesterol diet and increased tumor growth and development of metastasis (217–219). Dietary cholesterol, that represents only the 30% of total cholesterol in the human body is delivered to liver through chylomicrons, which are hydrolyzed to fatty acids and stored in adipose tissue (220). Once cholesterol arrives at hepatocytes, it is removed either in form of free cholesterol or converted in bile acids. Excess of cholesterol is converted into cholesterol esters and stored in hepatocytes (221). Accumulating evidence, based on metabolic mechanisms, highlights that tissues enriched of stored fatty acids could be more prone to be related with increased cancer risk (222, 223). Pelton and coworkers (224) demonstrated that high-cholesterol diet enhances the tumor growth of human breast cancer injected in a mouse model. In light of this consideration, the administration of low-cholesterol diet or ezetimibe slightly decreased the growth of tumors by a reduction of cholesterol levels (224). Moreover, it has been shown that the inhibition of the xanthine oxidase with a pharmacological inhibitor was able to reduce both tumor growth and metastasis in breast mouse model fed with high-cholesterol diet (225). Thus, it appears that a low-cholesterol diet could be a promising strategy to counteract tumor growth. Furthermore, combining the low-cholesterol diet with anticancer drugs could become an exploitable field in clinical therapy.

### Dysregulated Cholesterol Efflux

In physiological conditions, the excess of cellular cholesterol is removed from peripheral tissues through a process controlled by ATP binding cassette transporters, mainly ABCA1. In general, malignant cells show decreased levels of ABCA1, thereby promoting intracellular cholesterol storage. Indeed, ABCA1 deregulation leads to cholesterol accumulation in the mitochondrial compartment, which in turn supports the malignant transformation (32, 155). Moreover, higher levels of intracellular cholesterol directly affect the lipid composition of plasma membranes, as well as their physical properties: cholesterol enrichment increases the phospholipids’ degree of order in the bilayer while reducing its permeability, thereby promoting tumor resistance to membrane-active anticancer drugs (226). Peroxisome proliferator-activated receptors (PPAR) α and γ play a pivotal role in modulating both intracellular and extracellular cholesterol fluctuations (227, 228). Indeed, PPARα and PPARγ activation promotes LXR-mediated ABCA1 expression, thus inducing cholesterol efflux to the lipid-poor apolipoprotein A-I (229). Also, PPARα blocks cholesterol biosynthesis by inhibiting sterol regulatory element-binding protein 2 (SREBP-2) activity (230). Accordingly, PPARα and PPARγ are considered tumor suppressor genes which inhibit tumor progression (231). On the other hand, cholesterol integration within the plasma-membrane reduces malignant cell migration and metastatic potential. Specifically, increased plasma membrane-associated cholesterol reduces the fluidity of the bilayer, resulting in restricted cell motility and EMT, a critical event for the development of tumor metastasis (232). The variation in cholesterol content, in fact, affects membrane fluidity, permeability and rigidity, thus impacting several processes, such as invasion, migration or tumor development, growth and metastasis (233). In particular, different studies reported that increased cholesterol percentage deriving from higher *de novo* biosynthesis leads to enhanced rigidity and decreased fluidity, thus contributing to decreased cell mobility (234), or to altered membrane permeability, which is involved in altered cancer cell response to drug treatments (226). In line with this, variations in intracellular cholesterol levels mediated by ABCA1 overexpression drive the onset of EMT and the promotion of tumor invasiveness, whilst human solid tumors at advanced stages are characterized by high levels of ABCA1 expression (235). Therefore, metastatic cancer cells substantially reduce their cholesterol levels in the plasma membrane compartment by overexpressing ABCA1, which eventually mediates cholesterol efflux (232). Conversely, the development of primary tumors requires pro-oncogenic and survival-stimulatory signaling pathways, which are dependent or modulated by lipid rafts (114). Accumulations of cholesterol in lipid rafts induce the aberrant activation of tyrosine kinase receptors, such as IGF1 and HER2, as well as PI3K/AKT-mediated tumorigenic signaling (234, 236, 237).

#### Pharmacological Modulation of PPARα

Saidi and coworkers (144) tested fenofibrate, which is an agonist of PPARα, in Ishikawa endometrial cancer cells. The results showed inhibition of cell viability and apoptosis induction. Moreover, the combined use of fenofibrate and retinoic acid, which is an agonist of retinoid-X-receptor (RXR), enhanced the inhibition of cell proliferation (144). The mechanism of action of fenofibrate still remains unclear. In fact, fenofibrate reduced the cell proliferation of human hepatoma cells through the inhibition of Akt phosphorylation and not through a PPARα-dependent mechanism (145). Jan et al. (146) proposed metabolic reprogramming as the mechanism underpinning the anticancer effect of fenofibrate. In particular, this drug caused the reduction of oral cancer cell proliferation and activated the glycolysis pathway. Moreover, *in vivo* administration of fenofibrate in mice reduced the tumor growth (146). The antitumor effect of fenofibrate through the reprogramming of cancer metabolism is also confirmed in gastric carcinoma. In fact, the use of this drug reduced both *in vitro* cancer cell proliferation and *in vivo* tumor growth. In addition, Chen and coworkers (238) demonstrated that treating cells with fenofibrate causes mitochondrial dysfunction due to its accumulation too, suggesting also the PPARα involvement in mitochondria reprogramming (238). Thus, targeting PPARα could be an interesting tool for cancer treatment. Moreover, fenofibrate was also tested in combination with chemotherapeutic agents, suggesting that using the combined therapy could be a strategy to overcome drug resistance. In fact, treating prostate resistant cancer cells with fenofibrate is able to resensitize them to docetaxel (149).

### Enhanced Cholesterol Storage

The overload of free cholesterol inside the cell is extremely toxic (239). Therefore, in physiological conditions, the excess of free cholesterol is avoided by producing its esterified form, namely cholesteryl ester (240). Cholesteryl esters can be readily stored into lipid droplets, thus preventing the lipotoxic potential of free cholesterol (241). The accumulation of intracellular cholesterol is strongly oncogenic and represents a common hallmark of cancer (242, 243). For instance, the intracellular levels of cholesteryl esters and lipid droplets are substantially increased in breast cancer, leukemia, and glioblastoma (244–246). In colorectal cancer, lipid droplet-enriched malignant stem cells are characterized by increased clonogenic and tumorigenic potentials (247). Consistently, the cholesteryl ester-producing enzyme acetyltransferase ACAT1 is upregulated in many cancers, including hepatocellular carcinoma, castration-resistant prostate cancer, and pancreatic cancer, whilst its expression positively correlates with reduced overall survival and recurrence-free survival in adrenocortical carcinoma (126, 248–250). ACAT1 overexpression and cholesteryl esters enrichment play a dual role in promoting cancer progression. Higher levels of cholesterol esterification lead to decreased contents of free cholesterol, thereby protecting malignant cells from ER stress and apoptosis (126, 251). On the other hand, esterified cholesterol represents an intracellular source of cholesterol, which can be exploited by cancer cells when needed to fuel the malignant phenotype. Consistently, the cholesteryl ester-metabolizing enzyme lysosomal acid lipase (LAL) is upregulated in tumor tissues, thus providing malignant cells with ready-to-use free cholesterol (243, 252). PTEN deficiency drives cholesteryl esters accumulation in pancreatic cancer through the activation of the downstream PI3K/Akt/mTOR/SREBP signaling pathway; increased content of esterified cholesterol promotes tumorigenesis and metastatic potential (126). Similarly, cholesterol reservoirs are enriched in advanced and metastatic human prostate cancer, while nearly absent in healthy prostate, benign prostatic hyperplasia, and prostatic intraepithelial neoplasia. Accumulation of cholesteryl esters is triggered by PTEN loss, which in turn induces the expression of SREBP and LDLR *via* PI3K/AKT/mTOR, thus promoting ACAT1-mediated cholesterol storage in lipid droplets (243). Thanks to cholesterol esterification and subsequent accumulation, prostate cancer cells reduce the intracellular levels of free cholesterol, thereby avoiding free cholesterol lipotoxicity and maintaining SREPB-induced cholesterol biogenesis and uptake (62, 253). Moreover, increased contents of esterified cholesterol might fuel the development of castration-refractory prostate tumors by providing androgen precursors for *de novo* steroidogenesis (125, 254, 255). The excess of lipid droplets accumulated in tumor cells is the leading cause of enhanced cell proliferation and the responsible of cancer aggressiveness. Thus, it appears that targeting enhanced cholesterol storage could be an interesting tool in cancer therapy (113).

#### Pharmacological Modulation of ACAT-1

*ACAT-1* is overexpressed in two ER^-^ lines of human breast cancer, MDA-MB-231, and MDA-MB-436. Higher expression of this enzyme could be related to a higher cell proliferation rate (122). Treating cells with CP-113818, which is an ACAT-1 inhibitor, caused a reduction in cell proliferation and migration, suggesting the correlation mentioned above (122). Bitter melon extract, a natural ACAT-1 inhibitor, exerts antitumor effects towards breast cancer cells. Shim and coworkers (123) fed orthotopic mice models of MDA-MB-231 cells with this compound showing a reduction in tumor growth through cholesterol metabolism modulation (123). It has also been demonstrated that *ACAT-1* is a metabolic “tumor promoter”, since it is overexpressed in human breast cancer cells leading to tumor formation and lung metastasis (256). Cancer cells use this mitochondrial enzyme to recycle ketone acids into acetyl-CoA enhancing the ATP production. Ozsvari and coworkers (257) investigated ACAT-1 as a therapeutic target and the use of *in silico* drug design identified *mitoketoscins.* These molecules belong to a new therapeutic class of drugs that inhibits mitochondrial functions and ACAT-1 (257). Lo et al. (258) demonstrated that *ACAT-1* is overexpressed in MES-SA/Dx doxorubicin-resistant uterine sarcoma cancer cells compared to the sensitive counterpart, suggesting a correlation with drug resistance. Thus, the *ACAT-1* knock-down caused a decrease in cell viability, showing an important role of this enzyme in the onset of drug resistance (258). Lee and coworkers (124) developed avasimin, which is a nanoformulation containing avasimibe, an ACAT-1 inhibitor. They tested the formulation in different human cancer cell lines, showing that avasimin was able to reduce lipid droplets accumulation in PC3 prostate cancer cells. Concerning the effect on cell viability the nanoformulation was used to treat human PC3, MIA-PaCa2 pancreatic cancer cells, A549 lung cancer cells, and HCT116 colon cancer cells, showing a reduction in cell viability in all cell lines. Moreover, they evaluated *in vivo* the avasimin effect in PC3 and HCT116 cell xenograft mouse model. The results evidenced a decrease in both tumor growths after the avasimin intravenous treatment (124). Li et al. (126) demonstrated an overexpression of *ACAT-1* in MIA PaCa-2 and PANC-1 human pancreatic cancer cells compared to normal cells. Treating cells with avasimibe or genetic silencing of *ACAT-1* caused the block of cholesterol esterification that led to a reduction in cell invasion and migration. Results showed a higher sensitivity to the ACAT-1 inhibition of cancer cells compared to the normal counterpart. A xenograft mouse model injected with MIA PaCa-2 cells was treated with avasimibe and results showed a reduction in tumor growth, decreased metastatic lesions in lymph nodes and in liver compared to untreated mice (126). Moreover, it has been suggested an important role of cholesteryl ester in the development of metastasis. Thus, Lee and coworkers (125) tested avasimibe in PC-3M metastatic prostate cancer cell lines derived from PC-3 xenografts liver metastasis. The treatment showed a decrease in cell migration rate. In addition, when they treated PC-3M xenograft mice with avasimin a reduction in tumor growth and metastasis development were observed. Taken together, these data suggested an implication of cholesteryl ester in the development of metastasis in prostate cancer. ACAT-1 inhibition compromised Wnt/β-catenin signaling consequently overcoming metastasis formation (125). Li et al. (128) demonstrated a correlation between cholesterol metabolism and gemcitabine resistance, since it was found a higher accumulation of cholesteryl ester in gemcitabine-resistant pancreatic ductal adenocarcinoma cells compared to the sensitive counterpart. In addition, it has been demonstrated that Akt is implicated in cholesteryl ester accumulation. Treatment of resistant cells with avasimibe, an ACAT-1 inhibitor, caused a reduction in cell proliferation. Moreover, the combined treatment with gemcitabine and avasimibe showed synergic effect *in vitro* and resulted in decreasing tumor growth in *in vivo* xenograft mouse model injected with Mia PaCa-2 cells. Avasimibe treatment downregulates Akt contributing to resensitization of resistant cells (128). It has been demonstrated that the upregulation of *ACAT-1* is implicated in the development of metastasis in LLC Lewis lung cancer. Treatment of LLC cells with avasimibe caused a decrease in cell proliferation and migration. Moreover, avasimibe alone or in combination with cyclophosphamide was able to reduce both tumor growth and metastasis formation in xenograft mouse model (127).

### Oncogenic Signaling and Cholesterol Homeostasis

In physiological conditions, cholesterol homeostasis is maintained by sterol-sensitive systems, mainly SREBP2 and Liver X receptors (LXR). Oncogenic potential gaining and tumor suppressor activity loss in cancer cells deeply affect cholesterol metabolism. As a general rule, oncogenic pathways induce cholesterol biosynthesis and uptake, thus promoting increased intracellular levels of sterols, while tumor suppressor pathways lead to cholesterol lowering inside the cells (34). Indeed, the oncogenic *MYC* induces cholesterol biosynthesis by upregulating HMGCR expression, which is essential during oesophageal squamous cell carcinoma malignant transformation (259, 260). Similarly, aberrant EGFR oncogenic signaling is involved in SCAP-mediated SREBP-2 activation, thus promoting LDLR expression and subsequent cholesterol uptake (261, 262). In human hepatocellular carcinomas, the pro-oncogenic activity of c-FOS mediates LXRα downregulation, which leads to cholesterol retention and production of tumorigenic oxysterols (263). Oxysterols are oxygenated cholesterol metabolites which target and modulate the activity of many nuclear receptors, including LXRs, retinoid-related orphan receptors (RORs), as well as the Hedgehog signaling pathway (264, 265). Among them, 27-hydroxycholesterol (27HC) is an endogenous selective estrogen receptor modulator involved in breast and prostate cancers progression (266). 27HC promotes cell proliferation through *p53* inactivation, as well as cell migratory potential *via* Signal Transducer and Activator of Transcription-3 (STAT-3)-mediated MMP9 activation and subsequent EMT induction (267, 268). 27HC is also involved in tumor angiogenesis by inducing VEGF activation through ERα signaling or reactive oxygen species-mediated STAT-3 recruiting (269). Consistently, advanced breast cancers upregulate CYP27A1 while decreasing the expression of CYP7B1, thereby promoting 27HC accumulation (31, 270). Higher levels of intracellular cholesterol in cancer cells are determined by aberrant HMGCR activity, due to disrupted sterol-controlled feedback regulation or SREBP-mediated overexpression (271–273). In hypoxic tumor microenvironments, SREBPs and their downstream genes are strongly upregulated and support cell survival and tumor growth (274). The activity of SREBPs is promoted by many oncogenic signaling pathways, including PI3K/Akt and Ras/ERK, which eventually induce cholesterol biosynthesis and uptake, while inhibiting its ABCA1-mediated efflux (275–277). In line with this, tumor suppressors genes *p53* and *PTEN* increase cholesterol clearance by increasing ABCA1 activity, while reducing cholesterol absorption and accumulation (126, 278). Indeed, *PTEN* and *p53* loss induce PI3K/Akt signaling, thereby promoting LDLR-induced cholesterol uptake and subsequent formation of cholesteryl esters (243, 279, 280).

#### Pharmacological Modulation of LXR

Liver X receptors (LXR) are nuclear receptors involved in cholesterol metabolism. Targeting LXR could be a good strategy because its activation is able to modulate the cholesterol pathway. The consequence is decreased cholesterol levels into cells, causing limited cancer cell proliferation. As already explained above, LXR can be activated by endogenous ligands, such as oxysterols but also by agonists, such as T0901317 (130). Treating LNCaP human prostate cancer cells with LXR agonist T0901317 caused cell death through apoptosis. Moreover, the treatment with T0901317 in a xenograft mouse model injected with LNCaP cells was able to reduce tumor growth (129). It is known that increased levels of cholesterol activate Akt enhancing its phosphorylation besides improving tumor progression (281). Pommier and coworkers investigated the effect of LXR activation on cholesterol metabolism. T0901317 treatment was able to increase the expression of LXR target gene *Abcg1* and consequently causing a higher cholesterol efflux. Moreover, overexpression of *Abcg1* modulates reverse cholesterol transport causing cholesterol exhaustion in rafts and the inactivation of Akt signaling pathway (129). T0901317 showed anticancer properties also in ovarian cancer. In fact, treatment of ovarian cancer cells with this compound was able to inhibit cell proliferation and cause apoptosis (134). It was demonstrated that MCF-7 human breast cancer cells express LXR. Thus, treating these cells with the LXR synthetic agonist, T0901317, and the natural one, 22(*R*)-hydroxycholesterol, resulted in a reduction of cell proliferation besides both agonists caused cell death through apoptosis. In addition, T0901317 treatment was able to decrease intracellular cholesterol and LXR activation increased the expression of its target gene *Abcg1*in MCF-7 cells (130). Furthermore, it has been demonstrated that the combined therapy of T0901317 and gefitinib, an anticancer drug, was able to reduce cell and tumor growth both *in vitro* and *in vivo* in a lung cancer model (135). This agonist combined with sorafenib enhanced the antitumor effect of the chemotherapeutic agent in hepatocellular carcinoma. In fact, the activation of LXR blocks two pathways, MET and EGFR, avoiding their availability for lipid rafts and consequently enhancing the efflux of cholesterol (136). MCF-7, T-47D, SK-BR-3, or MDA-MB-231 human breast cancer cell lines, which are genetically different, expressed both LXR isoforms, LXR-α and LXR-β. Treating these cell lines with GW3965 LXR ligand caused a reduction in proliferation. Nguyen-Vu and coworkers correlated decreased cell proliferation with the downregulation of genes involved in cell growth. For example, they showed that the downregulation of E2F2, which is a transcription factor, caused a reduced proliferation of MCF-7 and T-47D ER^+^ cancer cells (137). GW3965 treatment inhibited cell proliferation in both human MCF-7 breast and SW480 colon cancer cell lines. Investigating the molecular mechanisms underlying this anti-proliferative effect, Hassan and coworkers (138) demonstrated that the activation of LXR caused the decrease of Akt phosphorylation leading to its inactivation (138). The expression of the isoform LXR-β was assessed in three different human pancreatic ductal adenocarcinoma cell lines, BxPC-3 and MIA-PaCa-2 and PANC-1. Treating cells with GW3965 increased the expression of the LXR target gene *Abca1*. Moreover, cell proliferation of human pancreatic ductal adenocarcinoma cell lines decreased after treatment with LXR agonist and the cell cycle was blocked (140).

The agonist GW3965 in combination with the standard chemotherapeutic agent gefitinib demonstrated synergic effect in resensitization of gefitinib-resistant lung cancer cells (141). Pencheva and her group (139) hypothesized that targeting LXR could be a strategy to block metastasis progression in melanoma. Treating melanoma cells with LXR agonists, GW3965 or T0901317, did not cause an impact on cell proliferation but affected cell invasion. In particular, the use of LXR agonists was able to block lung metastasis development and reduce brain metastasis progression in mouse melanoma models. In addition, it was also demonstrated that oral or diet administration of GW3965 to dacarbazine-resistant mice was able to strongly reduce melanoma tumor growth and that the combined treatment of LXR agonist with dacarbazine has proven to be more active compared to GW3965 alone. Moreover, the same agonist was able to reduce tumor growth in mouse melanoma models resistant to vemurafenib, and again, the combined treatment of GW3965 with vemurafenib had a higher effect compared to LXR alone (139). Zhang and coworkers (131) demonstrated that both LXR isoforms are expressed in murine B16F10 melanoma cells. When LXR is activated through the agonist T0901317 there was a decrease in melanoma cell proliferation and apoptosis through caspase-3 activation. Moreover, the treatment of mouse melanoma models with T0901317 reduced tumor growth. In order to confirm the involvement of LXR signaling in melanoma anti-tumor activity, the LXR target genes *Abca1* and *SREBF1* were checked confirming their increased expression in mice treated with T0901317 (131). Human multiple myeloma cells expressed both LXR isoforms, LXR-α and LXR-β. Treating cells with LXR ligand, 22(*R*)-hydroxycholesterol and the two agonists, GW3965 and T0901317, strongly increased the expression of two target genes, *Abca1* and *Abcg1*, while slightly the one of the target gene *SREBP-1c* (132). The Hedgehog (Hh) signaling pathway is a regulator of proliferation, differentiation and it has been linked to carcinogenesis (282). Agarwal and coworkers showed that activating LXR represents a strategy to inhibit Hh signaling pathway in human multiple myeloma cells. Moreover, they showed that treating cells with LXR agonists was able to inhibit clonogenic growth both *in vitro* and *in vivo* (132). SR9243 is a specific inverse agonist of LXR. Flaveny et al. (142) demonstrated that treatment of prostate, lung and colon cancer cells with SR9243 decreases cell proliferation, causes cell death through apoptosis and reduces tumor growth in mouse xenografts. Moreover, a combined treatment of SR9243 with cisplatin or 5^’^-fluorouracil sensitized cells to chemotherapeutic drugs. In particular, SR9243 treatment caused down-regulation of *GCK1*, *PFK2*, *PFK1*, and *LDH* Warburg genes, and decreased expression of *FASN*, *SREBP1-c*, and *SCD1* lipogenic gene both in *in vitro* and *in vivo* colon xenograft models (142). Similar results regarding lipid metabolism were obtained in clear renal cell carcinoma both *in vitro* and *in vivo* (143). SR9243 was also able to reduce the expression of LXR target gene *Abca1* involved in cholesterol transport (142). In addition, Wu et al. (143) tested the LXR agonist LXR623 in clear cell renal cell carcinoma demonstrating its role in decreasing cell proliferation and causing apoptosis. Considering that LXR is a transcription factor able to regulate the expression of different target genes, including those related to glycolysis and lipogenesis, targeting this receptor could represent a promising approach in cancer treatment. Kaneko and coworkers (133) demonstrated that LXR-α was expressed in human oral squamous cell carcinoma. Thus, activating LXR with T0901317 resulted in a reduction in cancer cell viability through the induction of the target gene *Abca1*. Moreover, SAS cells were injected in SCID mice and then they were treated with T0901317. The results evidenced a reduction in tumor growth after treatment (133).

The LXR agonist GW3965 was also used to target *LDLR* in glioblastoma. In fact, this pharmacological approach both inhibits the uptake of exogenous LDL and enhances the cholesterol excision from cells. Treating cancer cells with this drug induced apoptosis *in vitro* and reduced *in vivo* tumor growth (283).

#### Pharmacological Modulation of SREBP

Fatostatin is a non-sterol diarylthiazole derivative and a specific inhibitor of SREBP. The mechanism of action of this drug consists in binding the SREBP cleavage activating protein (SCAP), and consequently blocking cholesterol biosynthesis (150, 151). Targeting SREBP could be a new pharmacological approach for cancer treatment. Fatostatin showed antitumor effect in both androgen-responsive and androgen-nonresponsive prostate cancer cells by the *in vitro* inhibition of cell proliferation and cell cycle arrest. In addition, it was able to reduce *in vivo* tumor growth (150). Gholkar and coworkers (284) investigated the mechanism underlying the antitumor effect of fatostatin in different types of tumors, such as human breast and cervix cells, showing its ability to block the tubulin polymerization and arrest cells in mitosis (284). Fatostatin reduced cell viability in endometrial cancer (151, 152) and decreased the tumor growth in xenograft mice enhancing their survival rate (151). ER-positive breast cancer cells treated with fatostatin showed decreased cell viability and higher lipid accumulation. In particular, increased ceramides’ levels are strictly related to apoptosis. The xenograft volume decreased after treatment with fatostatin (153). Moreover, the combined treatment with tamoxifen resulted synergic in reducing both *in vitro* cell proliferation and *in vivo* tumor growth in breast cancer (154).

## Conclusions

**Figure 3:**
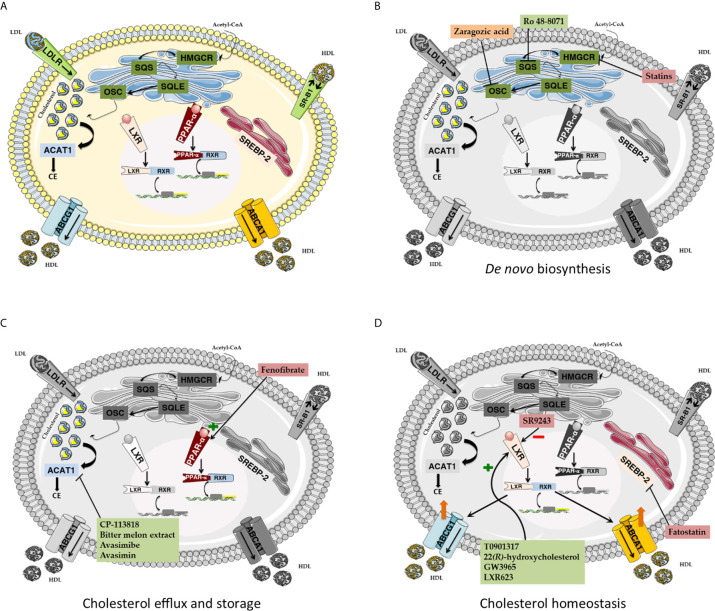
**(A)** Cholesterol metabolism*. De novo* cholesterol biosynthesis mainly relies on the activity of four key enzymes. HMGCR catalyzes the formation of mevalonate. Mevalonate is essential for farnesyl pyrophosphate biosynthesis, which is in turn exploited by SQS for squalene production. SQL converts squalene into its epoxydic form, which is eventually cyclized to lanosterol by OSC. Lastly, lanosterol is converted to cholesterol. HDL particles collect extrahepatic cholesterol and allow its cellular uptake by interacting with SR-B1. Alternatively, LDL-associated cholesterol can be captured and internalized in coated endocytic vesicles in a LDLR-mediated fashion. Intracellular cholesterol excess is converted into cholesteryl esters by ACAT1 and stored into lipid droplets. Cellular cholesterol efflux is mainly controlled by ABCA1 and ABCG1, two regulatory proteins belonging to the ATP-binding cassette transporter superfamily. Cellular cholesterol homeostasis is maintained by sterol-sensitive systems, such as SREBP2 and LXR. SREBP2-mediated adaptative response promotes cholesterol biosynthesis and uptake. Conversely, LXR promotes cholesterol excretion while impairing its uptake and production. PPARα activation promotes LXR-mediated ABCA1 expression and blocks cholesterol biosynthesis by inhibiting SREBP2. **(B)** Pharmacological targeting of *de novo* cholesterol biosynthesis pathway. Statins target and inhibit the activity of the rate-limiting enzyme HMGCR. Ro 48-8071 and Zaragozic acid act downstream of the mevalonate pathway, by inhibiting the activity of SQS and OSC, respectively. **(C)** Pharmacological targeting of cholesterol efflux and storage. The PPARα agonist Fenofibrate promotes PPARα-RXR interaction, thereby activating the PPARα signaling cascade. Both synthetic (CP-113818, Avasimibe, Avasimin) and natural (Bitter melon extract) inhibitors of ACAT-1 block cholesterol esterification and intracellular overload. **(D)** Pharmacological targeting of cholesterol homeostasis. LXR agonists, such as GW3965, T0901317, 22(R)-hydroxycholesterol and LXR623, can activate LXR signaling cascade, leading to increased cholesterol efflux and reduced cholesterol uptake. HMGCR, 3-hydroxy-3-methylglutaryl-CoA reductase; SQS, Squalene synthase; SQLE, Squalene epoxidase; OSC, 2,3-oxidosqualene cyclase; SR B1, scavenger receptor type B class 1; LDLR, LDL receptor; ACAT1, Acetyl-CoA Acetyltransferase 1. PPAR- α, peroxisome proliferator-activated receptor; LXR, liver X receptor; SREBP-2, sterol regulatory element-binding protein 2; ABCA1, ATP Binding Cassette Subfamily A Member 1; ABCG1, ATP Binding Cassette Subfamily G Member 1; HDL, High-density Lipoprotein; LDL, Low-density Lipoprotein.

This review examined the most relevant aspects of the metabolic reprogramming that have been observed in cancer focusing on cholesterol metabolism. Understanding the metabolic vulnerabilities of tumor tissues can help in the identification of new therapeutic targets in order to develop a better cancer treatment. The large amount of literature of the last decades provided overwhelming evidence of lipid and cholesterol metabolism alterations in cancer. High levels of cholesterol are essential to sustain fast tumor cell proliferation and the complex role of cholesterol in cancer development, progression, and susceptibility to chemotherapy is firmly established. Intracellular cholesterol levels can be regulated by *de novo* synthesis, reduced degradation, increased uptake or storage. This review summarizes the current knowledge regarding the alteration in all these aspects of cholesterol metabolism, highlighting the molecular targets and the possible pharmacological approaches that are currently under investigation (**Figure 3** and **Table 2**). Despite a large amount of *in vitro* and *in vivo* evidence suggesting the use of cholesterol-related drugs against cancer, the clinical translation is still limited (**Table 3**). To date, only statins and fenofibrate have resulted in clinical trials for cancer therapy showing promising results. Besides the treatment with a single agent, a common therapeutic strategy is the drug combination, which can affect simultaneously different pathways in cancers; thus, it is of interest to underline that the combination of conventional chemotherapeutic drugs with cholesterol-lowering agents is under investigation showing encouraging results.

A better understanding of the metabolic dependencies of tumors also provides new hints for therapeutic strategies in cancer therapy. Novel studies are focusing on the exploitation of lipid/cholesterol metabolic vulnerabilities of cancer to develop new drug delivery systems and strategies. Recent works make use of engineered lipids or adipocytes to deliver anticancer drugs to the tumors (285, 286). Moreover, LDL and HDL particles have been proposed as delivery systems for anticancer drugs. A work by Sobot et al. (287) proposed a chemical linkage of gemcitabine to squalene-moiety (the lipid precursors of cholesterol biosynthesis) assembled in nanoparticles. They showed that LDLR levels positively correlated with nanoparticle uptake and cytotoxic effect in cancer cells and in tumor-bearing mice (287). Mooberry and coworkers, instead, tested a formulation of paclitaxel encapsulated in synthetic/reconstituted high-density lipoprotein (rHDL). The increased uptake of anticancer drug is mediated by SR-B1, which is overexpressed in prostate cancer cells (288). Thus, conjugation of cholesterol moieties with anticancer drugs is an attractive approach (289) for cancer therapy, which can also improve the chemotherapy efficacy and reduce the cytotoxicity to normal cells.

The repurposing of cholesterol-lowering drugs for cancer therapy might be a promising approach to selectively affect cancer cells, highly dependent from cholesterol and to eventually improve the efficacy of conventional chemotherapy by affecting different signaling/metabolic pathways. A deep elucidation of cholesterol-linked metabolic vulnerabilities in cancers may offer new opportunities to develop new drug delivery strategies, allowing a more selective targeting of cancer cells, thus improving the quality of cancer therapy in patients.

## Author Contributions 

VC and ER conceived the review. IG and FG wrote the literature review. VC, MB, GO, TP-G, MD, and ER supervised and edited the review. All authors contributed to the article and approved the submitted version.

## Funding

IG, FG, VC, GO, and ER are supported by the University of Padova.

## Conflict of Interest

The authors declare that the research was conducted in the absence of any commercial or financial relationships that could be construed as a potential conflict of interest.

The reviewer AC declared a shared affiliation with the authors to the handling editor at the time of review.

## References

[1] Yoshida GJ. Metabolic Reprogramming: The Emerging Concept and Associated Therapeutic Strategies. J Exp Clin Cancer Res (2015) 34:111. 10.1186/s13046-015-0221-y 26445347 PMC4595070

[2] Ward PS Thompson CB. Metabolic Reprogramming: A Cancer Hallmark Even Warburg Did Not Anticipate. Cancer Cell (2012) 21:297–308. 10.1016/j.ccr.2012.02.014 22439925 PMC3311998

[3] Cocetta V Ragazzi E Montopoli M. Mitochondrial Involvement in Cisplatin Resistance. Int J Mol Sci (2019) 20:3384. 10.3390/ijms20143384 PMC6678541 31295873

[4] Zaal EA Berkers CR. The Influence of Metabolism on Drug Response in Cancer. Front Oncol (2018) 8:500. 10.3389/fonc.2018.00500 30456204 PMC6230982

[5] Desbats MA Giacomini I Prayer-Galetti T Montopoli M. Metabolic Plasticity in Chemotherapy Resistance. Front Oncol (2020) 10:281. 10.3389/fonc.2020.00281 32211323 PMC7068907

[6] Fendt S-M Frezza C Erez A. Targeting Metabolic Plasticity and Flexibility Dynamics for Cancer Therapy. Cancer Discov (2020) 10:1797–807. 10.1158/2159-8290.CD-20-0844 PMC7710573 33139243

[7] Giacomini I Ragazzi E Pasut G Montopoli M. The Pentose Phosphate Pathway and Its Involvement in Cisplatin Resistance. Int J Mol Sci (2020) 21:937. 10.3390/ijms21030937 PMC7036764 32023830

[8] Morandi A Indraccolo S. Linking Metabolic Reprogramming to Therapy Resistance in Cancer. Biochim Biophys Acta BBA - Rev Cancer (2017) 1868:1–6. 10.1016/j.bbcan.2016.12.004 28065746

[9] Cocetta V Ragazzi E Montopoli M. Links Between Cancer Metabolism and Cisplatin Resistance. Int Rev Cell Mol Biol (2020) 354:107–64. 10.1016/bs.ircmb.2020.01.005 32475471

[10] Phan LM Yeung S-CJ Lee M-H. Cancer Metabolic Reprogramming: Importance, Main Features, and Potentials for Precise Targeted Anti-Cancer Therapies. Cancer Biol Med (2014) 11:1–19. 10.7497/j.issn.2095-3941.2014.01.001 24738035 PMC3969803

[11] Olson RE. Discovery of the Lipoproteins, Their Role in Fat Transport and Their Significance as Risk Factors. J Nutr (1998) 128:439S–43S. 10.1093/jn/128.2.439S 9478044

[12] Schekman R. Discovery of the Cellular and Molecular Basis of Cholesterol Control. Proc Natl Acad Sci (2013) 110:14833–6. 10.1073/pnas.1312967110 PMC3773783 23975928

[13] Simons K Ikonen E. How Cells Handle Cholesterol. Science (2000) 290:1721–6. 10.1126/science.290.5497.1721 11099405

[14] Farese RV Herz J. Cholesterol Metabolism and Embryogenesis. Trends Genet (1998) 14:115–20. 10.1016/S0168-9525(97)01377-2 9540409

[15] Goldstein JL Brown MS. History of Discovery : The LDL Receptor. Arterioscler Thromb (2010) 29:431–8. 10.1161/ATVBAHA.108.179564.History PMC2740366 19299327

[16] Munro S. Lipid Rafts: Elusive or Illusive? Cell (2003) 115:377–88. 10.1016/S0092-8674(03)00882-1 14622593

[17] Simons K Ehehalt R. Cholesterol, Lipid Rafts, and Disease. J Clin Invest (2002) 110:597–603. 10.1172/JCI0216390 12208858 PMC151114

[18] Sezgin E Levental I Mayor S Eggeling C. The Mystery of Membrane Organization: Composition, Regulation and Roles of Lipid Rafts. Nat Rev Mol Cell Biol (2017) 18:361–74. 10.1038/nrm.2017.16 PMC5500228 28356571

[19] Simons K Ikonen E. Functional Rafts in Cell Membranes. Nature (1997) 387:569–72. 10.1038/42408 9177342

[20] Kolesnick R. The Therapeutic Potential of Modulating the Ceramide/Sphingomyelin Pathway. J Clin Invest (2002) 110:3–8. 10.1172/JCI16127 12093880 PMC151041

[21] van der Goot FG Harder T. Raft Membrane Domains: From a Liquid-Ordered Membrane Phase to a Site of Pathogen Attack. Semin Immunol (2001) 13:89–97. 10.1006/smim.2000.0300 11308292

[22] Head BP Patel HH Insel PA. Interaction of Membrane/Lipid Rafts With the Cytoskeleton: Impact on Signaling and Function. Biochim Biophys Acta (2014) 1838:532–45. 10.1016/j.bbamem.2013.07.018 PMC3867519 23899502

[23] Simons K Toomre D. Lipid Rafts and Signal Transduction. Nat Rev Mol Cell Biol (2000) 1:31–9. 10.1038/35036052 11413487

[24] Buhaescu I Izzedine H. Mevalonate Pathway: A Review of Clinical and Therapeutical Implications. Clin Biochem (2007) 40:575–84. 10.1016/j.clinbiochem.2007.03.016 17467679

[25] Shi Z Ruvkun G. The Mevalonate Pathway Regulates microRNA Activity in Caenorhabditis Elegans. Proc Natl Acad Sci USA (2012) 109:4568–73. 10.1073/pnas.1202421109 PMC3311396 22396595

[26] Berndt N Hamilton AD Sebti SM. Targeting Protein Prenylation for Cancer Therapy. Nat Rev Cancer (2011) 11:775–91. 10.1038/nrc3151 PMC4037130 22020205

[27] Hulce JJ Cognetta AB Niphakis MJ Tully SE Cravatt BF. Proteome-Wide Mapping of Cholesterol-Interacting Proteins in Mammalian Cells. Nat Methods (2013) . 10:259–64. 10.1038/nmeth.2368 PMC3601559 23396283

[28] Martins IJ Hone E Foster JK Sünram-Lea SI Gnjec A Fuller SJ. Apolipoprotein E, Cholesterol Metabolism, Diabetes, and the Convergence of Risk Factors for Alzheimer’s Disease and Cardiovascular Disease. Mol Psychiatry (2006) 11:721–36. 10.1038/sj.mp.4001854 16786033

[29] Van Der Kant R Goldstein LSB Ossenkoppele R. Amyloid-β-Independent Regulators of Tau Pathology in Alzheimer Disease. Nat Rev Neurosci (2020) 21:21–35. 10.1038/s41583-019-0240-3 31780819

[30] Haase CL Tybjærg-Hansen A Nordestgaard BG Frikke-Schmidt R. HDL Cholesterol and Risk of Type 2 Diabetes: A Mendelian Randomization Study. Diabetes (2015) 64:3328–33. 10.2337/db14-1603 25972569

[31] Silvente-Poirot S Poirot M. Cholesterol and Cancer, in the Balance. Science (2014) 343:1445–6. 10.1126/science.1252787 24675946

[32] Kuzu OF Noory MA Robertson GP. The Role of Cholesterol in Cancer. Cancer Res (2016) 76:2063–70. 10.1158/0008-5472.CAN-15-2613 PMC5813477 27197250

[33] Germain N Dhayer M Boileau M Fovez Q Kluza J Marchetti P. Lipid Metabolism and Resistance to Anticancer Treatment. Biology (2020) 9:474. 10.3390/biology9120474 PMC7766644 33339398

[34] Huang B Song B Xu C. Cholesterol Metabolism in Cancer: Mechanisms and Therapeutic Opportunities. Nat Metab (2020) 2:132–41. 10.1038/s42255-020-0174-0 32694690

[35] Ding X Zhang W Li S Yang H. The Role of Cholesterol Metabolism in Cancer. Am J Cancer Res (2019) 9:219–27. PMC6405981 30906624

[36] Cruz PMR Mo H McConathy WJ Sabnis N Lacko AG. The Role of Cholesterol Metabolism and Cholesterol Transport in Carcinogenesis: A Review of Scientific Findings, Relevant to Future Cancer Therapeutics. Front Pharmacol (2013) 4:119. 10.3389/fphar.2013.00119 24093019 PMC3782849

[37] Xu H Zhou S Tang Q Xia H Bi F. Cholesterol Metabolism: New Functions and Therapeutic Approaches in Cancer. Biochim Biophys Acta Rev Cancer (2020) 1874:188394. 10.1016/j.bbcan.2020.188394 32698040

[38] Repa JJ Mangelsdorf DJ. The Role of Orphan Nuclear Receptors in the Regulation of Cholesterol Homeostasis. Annu Rev Cell Dev Biol (2000) 16:459–81. 10.1146/annurev.cellbio.16.1.459 11031244

[39] Kruit JK Groen AK van Berkel TJ Kuipers F. Emerging Roles of the Intestine in Control of Cholesterol Metabolism. World J Gastroenterol (2006) 12:6429–39. 10.3748/wjg.v12.i40.6429 PMC4100631 17072974

[40] Luo J Yang H Song B-L. Mechanisms and Regulation of Cholesterol Homeostasis. Nat Rev Mol Cell Biol (2020) 21:225–45. 10.1038/s41580-019-0190-7 31848472

[41] Mo H Elson CE. Studies of the Isoprenoid-Mediated Inhibition of Mevalonate Synthesis Applied to Cancer Chemotherapy and Chemoprevention. Exp Biol Med (2004) 229:567–85. 10.1177/153537020422900701 15229351

[42] Ikonen E. Cellular Cholesterol Trafficking and Compartmentalization. Nat Rev Mol Cell Biol (2008) 9:125–38. 10.1038/nrm2336 18216769

[43] Singh P Saxena R Srinivas G Pande G Chattopadhyay A. Cholesterol Biosynthesis and Homeostasis in Regulation of the Cell Cycle. PloS One (2013) 8:e58833. 10.1371/journal.pone.0058833 23554937 PMC3598952

[44] Kandutsch AA Russell AE. Preputial Gland Tumor Sterols. 3. A Metabolic Pathway From Lanosterol to Cholesterol. J Biol Chem (1960) 235:2256–61. 10.1016/S0021-9258(18)64608-3 14404284

[45] Ačimovič J Goyal S Košir R Goličnik M Perše M Belič A. Cytochrome P450 Metabolism of the Post-Lanosterol Intermediates Explains Enigmas of Cholesterol Synthesis. Sci Rep (2016) 6:28462. 10.1038/srep28462 27334049 PMC4917857

[46] Jin U Park SJ Park SM. Cholesterol Metabolism in the Brain and Its Association With Parkinson’s Disease. Exp Neurobiol (2019) 28:554–67. 10.5607/en.2019.28.5.554 PMC6844833 31698548

[47] Goedeke L Fernández-Hernando C. Regulation of Cholesterol Homeostasis. Cell Mol Life Sci (2012) 69:915–30. 10.1007/s00018-011-0857-5 PMC11114919 22009455

[48] Jia L Betters JL Yu L. Niemann-Pick C1-Like 1 (NPC1L1) Protein in Intestinal and Hepatic Cholesterol Transport. Annu Rev Physiol (2011) 73:239–59. 10.1146/annurev-physiol-012110-142233 PMC3965667 20809793

[49] Betters JL Yu L. NPC1L1 and Cholesterol Transport. FEBS Lett (2010) 584:2740–7. 10.1016/j.febslet.2010.03.030 PMC2909875 20307540

[50] Nguyen TM Sawyer JK Kelley KL Davis MA Rudel LL. Cholesterol Esterification by ACAT2 is Essential for Efficient Intestinal Cholesterol Absorption: Evidence From Thoracic Lymph Duct Cannulation. J Lipid Res (2012) 53:95–104. 10.1194/jlr.M018820 22045928 PMC3243485

[51] Ko CW Qu J Black DD Tso P. Regulation of Intestinal Lipid Metabolism: Current Concepts and Relevance to Disease. Nat Rev Gastroenterol Hepatol (2020) 17:169–83. 10.1038/s41575-019-0250-7 32015520

[52] Ginsberg HN. Lipoprotein Physiology. Endocrinol Metab Clin North Am (1998) 27:503–19. 10.1016/s0889-8529(05)70023-2 9785050

[53] Ramasamy I. Recent Advances in Physiological Lipoprotein Metabolism. Clin Chem Lab Med (2014) 52:1695–727. 10.1515/cclm-2013-0358 23940067

[54] Holmes MV Ala-Korpela M. What Is “LDL Cholesterol”? Nat Rev Cardiol (2019) 16:197–8. 10.1038/s41569-019-0157-6 30700860

[55] Go GW Mani A. Low-Density Lipoprotein Receptor (LDLR) Family Orchestrates Cholesterol Homeostasis. Yale J Biol Med (2012) 85:19–28. 22461740 PMC3313535

[56] Luo X Cheng C Tan Z Li N Tang M Yang L. Emerging Roles of Lipid Metabolism in Cancer Metastasis. Mol Cancer (2017) 16:76. 10.1186/s12943-017-0646-3 28399876 PMC5387196

[57] Kwon HJ Abi-Mosleh L Wang ML Deisenhofer J Goldstein JL Brown MS. Structure of N-Terminal Domain of NPC1 Reveals Distinct Subdomains for Binding and Transfer of Cholesterol. Cell (2009) 137:1213–24. 10.1016/j.cell.2009.03.049 PMC2739658 19563754

[58] Li J Pfeffer SR. Lysosomal Membrane Glycoproteins Bind Cholesterol and Contribute to Lysosomal Cholesterol Export. eLife (2016) 5:e21635. 10.7554/eLife.21635 27664420 PMC5068966

[59] Ouimet M Barrett TJ Fisher EA. HDL and Reverse Cholesterol Transport. Circ Res (2019) 124:1505–18. 10.1161/CIRCRESAHA.119.312617 PMC6813799 31071007

[60] Brufau G Groen AK Kuipers F. Reverse Cholesterol Transport Revisited: Contribution of Biliary Versus Intestinal Cholesterol Excretion. Arterioscler Thromb Vasc Biol (2011) 31:1726–33. 10.1161/ATVBAHA.108.181206 21571685

[61] Röhrl C Stangl H. HDL Endocytosis and Resecretion. Biochim Biophys Acta (2013) 1831:1626–33. 10.1016/j.bbalip.2013.07.014 PMC3795453 23939397

[62] Chang TY Chang CCY Ohgami N Yamauchi Y. Cholesterol Sensing, Trafficking, and Esterification. Annu Rev Cell Dev Biol (2006) 22:129–57. 10.1146/annurev.cellbio.22.010305.104656 16753029

[63] Gelissen IC Harris M Rye KA Quinn C Brown AJ Kockx M. ABCA1 and ABCG1 Synergize to Mediate Cholesterol Export to Apoa-I. Arterioscler Thromb Vasc Biol (2006) 26:534–40. 10.1161/01.ATV.0000200082.58536.e1 16357317

[64] Ossoli A Pavanello C Calabresi L. High-Density Lipoprotein, Lecithin: Cholesterol Acyltransferase, and Atherosclerosis. Endocrinol Metab (2016) 31:223. 10.3803/EnM.2016.31.2.223 PMC4923405 27302716

[65] Chang TY Li BL Chang CCY Urano Y. Acyl-Coenzyme A:Cholesterol Acyltransferases. Am J Physiol Endocrinol Metab (2009) 297:E1–9. 10.1152/ajpendo.90926.2008 PMC2711667 19141679

[66] Petan T Jarc E Jusović M. Lipid Droplets in Cancer: Guardians of Fat in a Stressful World. Molecules (2018) 23:1941. 10.3390/molecules23081941 PMC6222695 30081476

[67] Miyazaki A Sakashita N Lee O Takahashi K Horiuchi S Hakamata H. Expression of ACAT-1 Protein in Human Atherosclerotic Lesions and Cultured Human Monocytes-Macrophages. Arterioscler Thromb Vasc Biol (1998) 18:1568–74. 10.1161/01.ATV.18.10.1568 9763528

[68] Chang CCY Sakashita N Ornvold K Lee O Chang ET Dong R. Immunological Quantitation and Localization of ACAT-1 and ACAT-2 in Human Liver and Small Intestine. J Biol Chem (2000) 275:28083–92. 10.1074/jbc.M003927200 10846185

[69] Joyce CW Shelness GS Davis MA Lee RG Skinner K Anderson RA. ACAT1 and ACAT2 Membrane Topology Segregates a Serine Residue Essential for Activity to Opposite Sides of the Endoplasmic Reticulum Membrane. Mol Biol Cell (2000) 11:3675–87. 10.1091/mbc.11.11.3675 PMC15029 11071899

[70] Steck TL Lange Y. Cell Cholesterol Homeostasis: Mediation by Active Cholesterol. Trends Cell Biol (2010) 20:680–7. 10.1016/j.tcb.2010.08.007 PMC2967630 20843692

[71] Wong J Quinn CM Brown AJ. SREBP-2 Positively Regulates Transcription of the Cholesterol Efflux Gene, ABCA1, by Generating Oxysterol Ligands for LXR. Biochem J (2006) 400:485–91. 10.1042/BJ20060914 PMC1698594 16901265

[72] Brown MS Radhakrishnan A Goldstein JL. Retrospective on Cholesterol Homeostasis: The Central Role of Scap. Annu Rev Biochem (2018) 87:783–807. 10.1146/annurev-biochem-062917-011852 28841344 PMC5828883

[73] Nagoshi E Yoneda Y. Dimerization of Sterol Regulatory Element-Binding Protein 2 Via the Helix-Loop-Helix-Leucine Zipper Domain is a Prerequisite for Its Nuclear Localization Mediated by Importin β. Mol Cell Biol (2001) 21:2779–89. 10.1128/MCB.21.8.2779-2789.2001 PMC86908 11283257

[74] Zhao X Yang F. Regulation of SREBP-Mediated Gene Expression. Acta Biophys Sin (2012) 28:287. 10.3724/SP.J.1260.2012.20034 PMC3667598 23730104

[75] Radhakrishnan A Sun LP Kwon HJ Brown MS Goldstein JL. Direct Binding of Cholesterol to the Purified Membrane Region of SCAP: Mechanism for a Sterol-Sensing Domain. Mol Cell (2004) 15:259–68. 10.1016/j.molcel.2004.06.019 15260976

[76] Howe V Sharpe LJ Prabhu AV Brown AJ. New Insights Into Cellular Cholesterol Acquisition: Promoter Analysis of Human HMGCR and SQLE , Two Key Control Enzymes in Cholesterol Synthesis. Biochim Biophys Acta Mol Cell Biol Lipids (2017) 1862:647–57. 10.1016/j.bbalip.2017.03.009 28342963

[77] Horton JD Goldstein JL Brown MS. SREBPs: Activators of the Complete Program of Cholesterol and Fatty Acid Synthesis in the Liver. J Clin Invest (2002) 109:1125–31. 10.1172/JCI0215593 PMC150968 11994399

[78] Theesfeld CL Pourmand D Davis T Garza RM Hampton RY. The Sterol-Sensing Domain (SSD) Directly Mediates Signal-Regulated Endoplasmic Reticulum-Associated Degradation (ERAD) of 3-Hydroxy-3-Methylglutaryl (HMG)-CoA Reductase Isozyme Hmg2. J Biol Chem (2011) 286:26298–307. 10.1074/jbc.M111.244798 PMC3143592 21628456

[79] Clarke PR Hardie DG. Regulation of HMG-CoA Reductase: Identification of the Site Phosphorylated by the AMP-Activated Protein Kinase In Vitro and in Intact Rat Liver. EMBO J (1990) 9:2439–46. 10.1002/j.1460-2075.1990.tb07420.x PMC552270 2369897

[80] Alrefai WA Annaba F Sarwar Z Dwivedi A Saksena S Singla A. Modulation of Human Niemann-Pick C1-Like 1 Gene Expression by Sterol: Role of Sterol Regulatory Element Binding Protein 2. Am J Physiol Gastrointest Liver Physiol (2007) 292:G369–376. 10.1152/ajpgi.00306.2006 17008555

[81] Kotzka J Müller-Wieland D Roth G Kremer L Munck M Schürmann S. Sterol Regulatory Element Binding Proteins (SREBP)-1a and SREBP-2 are Linked to the MAP-kinase Cascade. J Lipid Res (2000) 41:99–108. 10.1016/S0022-2275(20)32079-4 10627507

[82] Sato R Inoue J Kawabe Y Kodama T Takano T Maeda M. Sterol-Dependent Transcriptional Regulation of Sterol Regulatory Element-binding Protein-2. J Biol Chem (1996) 271:26461–4. 10.1074/jbc.271.43.26461 8900111

[83] Radhakrishnan SK den Besten W Deshaies RJ. p97-dependent Retrotranslocation and Proteolytic Processing Govern Formation of Active Nrf1 Upon Proteasome Inhibition. eLife (2014) 3:e01856. 10.7554/eLife.01856 24448410 PMC3896944

[84] Wang B Tontonoz P. Liver X Receptors in Lipid Signalling and Membrane Homeostasis. Nat Rev Endocrinol (2018) 14:452–63. 10.1038/s41574-018-0037-x PMC6433546 29904174

[85] Peet DJ Turley SD Ma W Janowski BA Lobaccaro JM Hammer RE. Cholesterol and Bile Acid Metabolism Are Impaired in Mice Lacking the Nuclear Oxysterol Receptor LXR Alpha. Cell (1998) 93:693–704. 10.1016/s0092-8674(00)81432-4 9630215

[86] Yvan-Charvet L Wang N Tall AR. Role of HDL, ABCA1, and ABCG1 Transporters in Cholesterol Efflux and Immune Responses. Arterioscler Thromb Vasc Biol (2010) 30:139–43. 10.1161/ATVBAHA.108.179283 PMC2812788 19797709

[87] Venkateswaran A Laffitte BA Joseph SB Mak PA Wilpitz DC Edwards PA. Control of Cellular Cholesterol Efflux by the Nuclear Oxysterol Receptor LXR Alpha. Proc Natl Acad Sci USA (2000) 97:12097–102. 10.1073/pnas.200367697 PMC17300 11035776

[88] Duval C Touche V Tailleux A Fruchart JC Fievet C Clavey V. Niemann–Pick C1 Like 1 Gene Expression is Down-Regulated by LXR Activators in the Intestine. Biochem Biophys Res Commun (2006) 340:1259–63. 10.1016/j.bbrc.2005.12.137 16414355

[89] Zelcer N Hong C Boyadjian R Tontonoz P. LXR Regulates Cholesterol Uptake Through Idol-Dependent Ubiquitination of the LDL Receptor. Science (2009) 325:100–4. 10.1126/science.1168974 PMC2777523 19520913

[90] Zhang L Reue K Fong LG Young SG Tontonoz P. Feedback Regulation of Cholesterol Uptake by the LXR–IDOL–LDLR Axis. Arterioscler Thromb Vasc Biol (2012) 32:2541–6. 10.1161/ATVBAHA.112.250571 PMC4280256 22936343

[91] Stancu C Sima A. Statins: Mechanism of Action and Effects. J Cell Mol Med (2001) 5:378–87. 10.1111/j.1582-4934.2001.tb00172.x PMC6740083 12067471

[92] Bedi O Dhawan V Sharma PL Kumar P. Pleiotropic Effects of Statins: New Therapeutic Targets in Drug Design. Naunyn Schmiedebergs Arch Pharmacol (2016) 389:695–712. 10.1007/s00210-016-1252-4 27146293

[93] Endo A. A Historical Perspective on the Discovery of Statins. Proc Jpn Acad Ser B (2010) 86:484–93. 10.2183/pjab.86.484 PMC3108295 20467214

[94] Davies JT Delfino SF Feinberg CE Johnson MF Nappi VL Olinger JT. Current and Emerging Uses of Statins in Clinical Therapeutics: A Review. Lipid Insights (2016) 9:13–29. 10.4137/LPI.S37450 27867302 PMC5110224

[95] Ramkumar S Raghunath A Raghunath S. Statin Therapy: Review of Safety and Potential Side Effects. Acta Cardiol Sin (2016) 32:631–9. 10.6515/ACS20160611A PMC5126440 27899849

[96] Liu A Wu Q Guo J Ares I Rodríguez J-L Martínez-Larrañaga MR. Statins: Adverse Reactions, Oxidative Stress and Metabolic Interactions. Pharmacol Ther (2019) 195:54–84. 10.1016/j.pharmthera.2018.10.004 30321555

[97] Ray KK Bays HE Catapano AL Lalwani ND Bloedon LT Sterling LR. Clear Harmony Trial. Safety and Efficacy of Bempedoic Acid to Reduce LDL Cholesterol. N Engl J Med (2019) 380:1022–32. 10.1056/NEJMoa1803917 30865796

[98] Fazio S Linton MF. The Role of Fibrates in Managing Hyperlipidemia: Mechanisms of Action and Clinical Efficacy. Curr Atheroscler Rep (2004) 6:148–57. 10.1007/s11883-004-0104-8 15023300

[99] Backes JM Gibson CA Ruisinger JF Moriarty PM. Fibrates: What Have We Learned in the Past 40 Years? Pharmacotherapy (2007) 27:412–24. 10.1592/phco.27.3.412 17316152

[100] Laganà A Vitale S Nigro A Sofo V Salmeri F Rossetti P. Pleiotropic Actions of Peroxisome Proliferator-Activated Receptors (PPARs) in Dysregulated Metabolic Homeostasis, Inflammation and Cancer: Current Evidence and Future Perspectives. Int J Mol Sci (2016) 17:999. 10.3390/ijms1707099933 PMC4964375 27347932

[101] Phan BA Dayspring TD Toth PP. Ezetimibe Therapy: Mechanism of Action and Clinical Update. Vasc Health Risk Manag (2012) 8:415–27. 10.2147/VHRM.S33664 PMC3402055 22910633

[102] Florentin M Liberopoulos EN Elisaf MS. Ezetimibe-Associated Adverse Effects: What the Clinician Needs to Know: Ezetimibe and Side Effects. Int J Clin Pract (2007) 62:88–96. 10.1111/j.1742-1241.2007.01592.x 18173814

[103] Corsini A Windler E Farnier M. Colesevelam Hydrochloride: Usefulness of a Specifically Engineered Bile Acid Sequestrant for Lowering LDL-Cholesterol. Eur J Cardiovasc Prev Rehabil (2009) 16:1–9. 10.1097/HJR.0b013e32831215db 19237992

[104] Scaldaferri F Pizzoferrato M Ponziani FR Gasbarrini G Gasbarrini A. Use and Indications of Cholestyramine and Bile Acid Sequestrants. Intern Emerg Med (2013) 8:205–10. 10.1007/s11739-011-0653-0 21739227

[105] Gouni-Berthold I Berthold HK. Mipomersen and Lomitapide: Two New Drugs for the Treatment of Homozygous Familial Hypercholesterolemia. Atheroscler Suppl (2015) 18:28–34. 10.1016/j.atherosclerosissup.2015.02.005 25936301

[106] Agarwala A Jones P Nambi V. The Role of Antisense Oligonucleotide Therapy in Patients With Familial Hypercholesterolemia: Risks, Benefits, and Management Recommendations. Curr Atheroscler Rep (2015) 17:467. 10.1007/s11883-014-0467-4 25398643

[107] Parhofer K. Mipomersen: Evidence-Based Review of its Potential in the Treatment of Homozygous and Severe Heterozygous Familial Hypercholesterolemia. Core Evid (2012) 7:29–38. 10.2147/CE.S25239 22701100 PMC3373191

[108] Goulooze SC Cohen AF Rissmann R. Lomitapide: New Drug Mechanisms: Lomitapide. Br J Clin Pharmacol (2015) 80:179–81. 10.1111/bcp.12612 PMC4541964 25702706

[109] Chaudhary R Garg J Shah N Sumner A. PCSK9 Inhibitors: A New Era of Lipid Lowering Therapy. World J Cardiol (2017) 9:76. 10.4330/wjc.v9.i2.76 28289523 PMC5329749

[110] Knopp RH. Drug Treatment of Lipid Disorders. N Engl J Med (1999) 341:498–511. 10.1056/NEJM199908123410707 10441607

[111] Gorin A Gabitova L Astsaturov I. Regulation of Cholesterol Biosynthesis and Cancer Signaling. Curr Opin Pharmacol (2012) 12:710–6. 10.1016/j.coph.2012.06.011 PMC3504641 22824431

[112] Kuzu OF Gowda R Noory MA Robertson GP. Modulating Cancer Cell Survival by Targeting Intracellular Cholesterol Transport. Br J Cancer (2017) 117:513–24. 10.1038/bjc.2017.200 PMC5558686 28697173

[113] Gu L Saha ST Thomas J Kaur M. Targeting Cellular Cholesterol for Anticancer Therapy. FEBS J (2019) 286:4192–208. 10.1111/febs.15018 31350867

[114] Li YC Park MJ Ye S-K Kim C-W Kim Y-N. Elevated Levels of Cholesterol-Rich Lipid Rafts in Cancer Cells Are Correlated With Apoptosis Sensitivity Induced by Cholesterol-Depleting Agents. Am J Pathol (2006) 168:1107–18. 10.2353/ajpath.2006.050959 PMC1606567 16565487

[115] Liu Z Liu X Liu S Cao Q. Cholesterol Promotes the Migration and Invasion of Renal Carcinoma Cells by Regulating the KLF5/miR-27a/FBXW7 Pathway. Biochem Biophys Res Commun (2018) 502:69–75. 10.1016/j.bbrc.2018.05.122 29782853

[116] Liang Y Besch-Williford C Aebi JD Mafuvadze B Cook MT Zou X. Cholesterol Biosynthesis Inhibitors as Potent Novel Anti-Cancer Agents: Suppression of Hormone-Dependent Breast Cancer by the Oxidosqualene Cyclase Inhibitor RO 48-8071. Breast Cancer Res Treat (2014) 146:51–62. 10.1007/s10549-014-2996-5 24878988 PMC11121502

[117] Grinter SZ Liang Y Huang S-Y Hyder SM Zou X. An Inverse Docking Approach for Identifying New Potential Anti-Cancer Targets. J Mol Graph Model (2011) 29:795–9. 10.1016/j.jmgm.2011.01.002 PMC3068237 21315634

[118] Maione F Oliaro-Bosso S Meda C Di Nicolantonio F Bussolino F Balliano G. The Cholesterol Biosynthesis Enzyme Oxidosqualene Cyclase is a New Target to Impair Tumour Angiogenesis and Metastasis Dissemination. Sci Rep (2015) 5:9054. 10.1038/srep09054 25761781 PMC4357009

[119] Liang Y Mafuvadze B Aebi JD Hyder SM. Cholesterol Biosynthesis Inhibitor RO 48-8071 Suppresses Growth of Hormone-Dependent and Castration-Resistant Prostate Cancer Cells. OncoTargets Ther (2016) 9:3223–32. 10.2147/OTT.S105725 PMC4892832 27313468

[120] Brusselmans K Timmermans L Van de Sande T Van Veldhoven PP Guan G Shechter I. Squalene Synthase, a Determinant of Raft-Associated Cholesterol and Modulator of Cancer Cell Proliferation. J Biol Chem (2007) 282:18777–85. 10.1074/jbc.M611763200 17483544

[121] Lanterna C Musumeci A Raccosta L Corna G Moresco M Maggioni D. The Administration of Drugs Inhibiting Cholesterol/Oxysterol Synthesis is Safe and Increases the Efficacy of Immunotherapeutic Regimens in Tumor-Bearing Mice. Cancer Immunol Immunother (2016) 65:1303–15. 10.1007/s00262-016-1884-8 PMC11029546 27520505

[122] Antalis CJ Arnold T Rasool T Lee B Buhman KK Siddiqui RA. High ACAT1 Expression in Estrogen Receptor Negative Basal-Like Breast Cancer Cells Is Associated With LDL-induced Proliferation. Breast Cancer Res Treat (2010) 122:661–70. 10.1007/s10549-009-0594-8 19851860

[123] Shim SH Sur S Steele R Albert CJ Huang C Ford DA. Disrupting Cholesterol Esterification by Bitter Melon Suppresses Triple-Negative Breast Cancer Cell Growth. Mol Carcinog (2018) 57:1599–607. 10.1002/mc.22882 30074275

[124] Lee SSY Li J Tai JN Ratliff TL Park K Cheng JX. Avasimibe Encapsulated in Human Serum Albumin Blocks Cholesterol Esterification for Selective Cancer Treatment. ACS Nano (2015) 9:2420–32. 10.1021/nn504025a PMC5909415 25662106

[125] Lee HJ Li J Vickman RE Li J Liu R Durkes AC. Cholesterol Esterification Inhibition Suppresses Prostate Cancer Metastasis by Impairing the Wnt/β-Catenin Pathway. Mol Cancer Res (2018) 16:974–85. 10.1158/1541-7786.MCR-17-0665 PMC5984676 29545473

[126] Li J Gu D Lee SSY Song B Bandyopadhyay S Chen S. Abrogating Cholesterol Esterification Suppresses Growth and Metastasis of Pancreatic Cancer. Oncogene (2016) 35:6378–88. 10.1038/onc.2016.168 PMC5093084 27132508

[127] Bi M Qiao X Zhang H Wu H Gao Z Zhou H. Effect of Inhibiting ACAT−1 Expression on the Growth and Metastasis of Lewis Lung Carcinoma. Oncol Lett (2019) 18:1548–56. 10.3892/ol.2019.10427 PMC6607388 31423222

[128] Li J Qu X Tian J Zhang JT Cheng JX. Cholesterol Esterification Inhibition and Gemcitabine Synergistically Suppress Pancreatic Ductal Adenocarcinoma Proliferation. PloS One (2018) 13:e0193318. 10.1371/journal.pone.0193318 29489864 PMC5831104

[129] Pommier AJC Alves G Viennois E Bernard S Communal Y Sion B. Liver X Receptor Activation Downregulates AKT Survival Signaling in Lipid Rafts and Induces Apoptosis of Prostate Cancer Cells. Oncogene (2010) 29:2712–23. 10.1038/onc.2010.30 20190811

[130] Roz AE Bard JM Huvelin JM Nazih H. LXR Agonists and ABCG1-dependent Cholesterol Efflux in MCF-7 Breast Cancer Cells: Relation to Proliferation and Apoptosis. Anticancer Res (2012) 32:3007–13. 22753765

[131] Zhang W Jiang H Zhang J Zhang Y Liu A Zhao Y. Liver X Receptor Activation Induces Apoptosis of Melanoma Cell Through Caspase Pathway. Cancer Cell Int (2014) 14:16. 10.1186/1475-2867-14-16 24564864 PMC3941804

[132] Agarwal JR Wang Q Tanno T Rasheed Z Merchant A Ghosh N. Activation of Liver X Receptors Inhibits Hedgehog Signaling, Clonogenic Growth, and Self-Renewal in Multiple Myeloma. Mol Cancer Ther (2014) 13:1873–81. 10.1158/1535-7163.MCT-13-0997 PMC4182725 24807964

[133] Kaneko T Kanno C Ichikawa-Tomikawa N Kashiwagi K Yaginuma N Ohkoshi C. Liver X Receptor Reduces Proliferation of Human Oral Cancer Cells by Promoting Cholesterol Efflux Via Up-Regulation of ABCA1 Expression. Oncotarget (2015) 6:33345–57. 10.18632/oncotarget.5428 PMC4741770 26452260

[134] Rough JJ Monroy MA Yerrum S Daly JM. Anti-Proliferative Effect of LXR Agonist T0901317 in Ovarian Carcinoma Cells. J Ovarian Res (2010) 3:13. 10.1186/1757-2215-3-13 20504359 PMC2890636

[135] Lou R Cao H Dong S Shi C Xu X Ma R. Liver X Receptor Agonist T0901317 Inhibits the Migration and Invasion of Non-Small-Cell Lung Cancer Cells *In Vivo* and In Vitro. Anticancer Drugs (2019) 30:495–500. 10.1097/CAD.0000000000000758 30724772 PMC6485493

[136] Shao W Zhu W Lin J Luo M Lin Z Lu L. Liver X Receptor Agonism Sensitizes a Subset of Hepatocellular Carcinoma to Sorafenib by Dual-Inhibiting MET and EGFR. Neoplasia (2020) 22:1–9. 10.1016/j.neo.2019.08.002 31751859 PMC6911865

[137] Nguyen-Vu T Vedin LL Liu K Jonsson P Lin JZ Candelaria NR. Liver × Receptor Ligands Disrupt Breast Cancer Cell Proliferation Through an E2F-Mediated Mechanism. Breast Cancer Res (2013) 15:R51. 10.1186/bcr3443 23809258 PMC4053202

[138] Hassan TS Paniccia A Russo V Steffensen KR. LXR Inhibits Proliferation of Human Breast Cancer Cells Through the PI3K-Akt Pathway. Nucl Recept Res (2015) 2:1–10. 10.11131/2015/101154

[139] Pencheva N Buss CG Posada J Merghoub T Tavazoie SF. Broad-Spectrum Therapeutic Suppression of Metastatic Melanoma Through Nuclear Hormone Receptor Activation. Cell (2014) 156:986–1001. 10.1016/j.cell.2014.01.038 24581497

[140] Candelaria NR Addanki S Zheng J Nguyen-Vu T Karaboga H Dey P. Antiproliferative Effects and Mechanisms of Liver X Receptor Ligands in Pancreatic Ductal Adenocarcinoma Cells. PloS One (2014) 9:e106289. 10.1371/journal.pone.0106289 25184494 PMC4153644

[141] Wang Q Shen B Qin X Liu S Feng J. Akt/mTOR and AMPK Signaling Pathways are Responsible for Liver X Receptor Agonist GW3965-Enhanced Gefitinib Sensitivity in Non-Small Cell Lung Cancer Cell Lines. Transl Cancer Res (2019) 8:66–76. 10.21037/tcr.2018.12.34 PMC8797756 35116735

[142] Flaveny CA Griffett K El-Gendy BE-DM Kazantzis M Sengupta M Amelio AL. Broad Anti-tumor Activity of a Small Molecule That Selectively Targets the Warburg Effect and Lipogenesis. Cancer Cell (2015) 28:42–56. 10.1016/j.ccell.2015.05.007 26120082 PMC4965273

[143] Wu G Wang Q Xu Y Li J Zhang H Qi G. Targeting the Transcription Factor Receptor LXR to Treat Clear Cell Renal Cell Carcinoma: Agonist or Inverse Agonist? Cell Death Dis (2019) 10:416. 10.1038/s41419-019-1654-6 31138790 PMC6538631

[144] Saidi SA Holland CM Charnock-Jones DS Smith SK. *In Vitro* and *In Vivo* Effects of the PPAR-Alpha Agonists Fenofibrate and Retinoic Acid in Endometrial Cancer. Mol Cancer (2006) 5:13. 10.1186/1476-4598-5-13 16569247 PMC1475879

[145] Yamasaki D Kawabe N Nakamura H Tachibana K Ishimoto K Tanaka T. Fenofibrate Suppresses Growth of the Human Hepatocellular Carcinoma Cell Via PPARα-Independent Mechanisms. Eur J Cell Biol (2011) 90:657–64. 10.1016/j.ejcb.2011.02.005 21514001

[146] Jan CI Tsai MH Chiu CF Huang YP Liu CJ Chang NW. Fenofibrate Suppresses Oral Tumorigenesis Via Reprogramming Metabolic Processes: Potential Drug Repurposing for Oral Cancer. Int J Biol Sci (2016) 12:786–98. 10.7150/ijbs.13851 PMC4910598 27313493

[147] Chen X Chen S Yu D. Metabolic Reprogramming of Chemoresistant Cancer Cells and the Potential Significance of Metabolic Regulation in the Reversal of Cancer Chemoresistance. Metabolites (2020) 10:289. 10.3390/metabo10070289 PMC7408410 32708822

[148] Robison NJ Campigotto F Chi SN Manley PE Turner CD Zimmerman MA. A Phase II Trial of a Multi-Agent Oral Antiangiogenic (Metronomic) Regimen in Children With Recurrent or Progressive Cancer. Pediatr Blood Cancer (2014) 61:636–42. 10.1002/pbc.24794 PMC4285784 24123865

[149] Luty M Piwowarczyk K Łabędź-Masłowska A Wróbel T Szczygieł M Catapano J. Fenofibrate Augments the Sensitivity of Drug-Resistant Prostate Cancer Cells to Docetaxel. Cancers (2019) 11:77. 10.3390/cancers11010077 PMC6356694 30641904

[150] Li X Chen YT Hu P Huang WC. Fatostatin Displays High Antitumor Activity in Prostate Cancer by Blocking Srebp-Regulated Metabolic Pathways and Androgen Receptor Signaling. Mol Cancer Ther (2014) 13:855–66. 10.1158/1535-7163.MCT-13-0797 PMC4084917 24493696

[151] Yao L Chen S Li W. Fatostatin Inhibits the Development of Endometrial Carcinoma in Endometrial Carcinoma Cells and a Xenograft Model by Targeting Lipid Metabolism. Arch Biochem Biophys (2020) 684:108327. 10.1016/j.abb.2020.108327 32142890

[152] Gao S Shi Z Li X Li W Wang Y Liu Z. Fatostatin Suppresses Growth and Enhances Apoptosis by Blocking SREBP-Regulated Metabolic Pathways in Endometrial Carcinoma. Oncol Rep (2018) 39:1919–29. 10.3892/or.2018.6265 29436682

[153] Brovkovych V Izhar Y Danes JM Dubrovskyi O Sakallioglu IT Morrow LM. Fatostatin Induces Pro- and Anti-Apoptotic Lipid Accumulation in Breast Cancer. Oncogenesis (2018) 7:66. 10.1038/s41389-018-0076-0 30140005 PMC6107643

[154] Liu Y Zhang N Zhang H Wang L Duan Y Wang X. Fatostatin in Combination With Tamoxifen Induces Synergistic Inhibition in ER-Positive Breast Cancer. Drug Des Devel Ther (2020) 14:3535–45. 10.2147/DDDT.S253876 PMC7457819 32921987

[155] Smith B Land H. Anticancer Activity of the Cholesterol Exporter ABCA1 Gene. Cell Rep (2012) 2:580–90. 10.1016/j.celrep.2012.08.011 PMC3462268 22981231

[156] Ehmsen S Pedersen MH Wang G Terp MG Arslanagic A Hood BL. Increased Cholesterol Biosynthesis Is a Key Characteristic of Breast Cancer Stem Cells Influencing Patient Outcome. Cell Rep (2019) 27:3927–38. 10.1016/j.celrep.2019.05.104 31242424

[157] Chushi L Wei W Kangkang X Yongzeng F Ning X Xiaolei C. HMGCR is Up-Regulated in Gastric Cancer and Promotes the Growth and Migration of the Cancer Cells. Gene (2016) 587:42–7. 10.1016/j.gene.2016.04.029 27085483

[158] Ashida S Kawada C Inoue K. Stromal Regulation of Prostate Cancer Cell Growth by Mevalonate Pathway Enzymes HMGCS1 and HMGCR. Oncol Lett (2017) 14:6533–42. 10.3892/ol.2017.7025 PMC5686443 29163687

[159] Wong W-LW Dimitroulakos J Minden M Penn L. HMG-CoA Reductase Inhibitors and the Malignant Cell: The Statin Family of Drugs as Triggers of Tumor-Specific Apoptosis. Leukemia (2002) 16:508–19. 10.1038/sj.leu.2402476 11960327

[160] Goldstein JL Brown MS. Regulation of the Mevalonate Pathway. Nature (1990) 343:425–30. 10.1038/343425a0 1967820

[161] Bonetti PO Lerman LO Napoli C Lerman A. Statin Effects Beyond Lipid Lowering—are They Clinically Relevant? Eur Heart J (2003) 24:225–48. 10.1016/S0195-668X(02)00419-0 12590901

[162] Gimple RC Wang X. RAS: Striking at the Core of the Oncogenic Circuitry. Front Oncol (2019) 9:965. 10.3389/fonc.2019.00965 31681559 PMC6798062

[163] Kaymak I Maier CR Schmitz W Campbell AD Dankworth B Ade CP. Mevalonate Pathway Provides Ubiquinone to Maintain Pyrimidine Synthesis and Survival in P53-Deficient Cancer Cells Exposed to Metabolic Stress. Cancer Res (2020) 80:189–203. 10.1158/0008-5472.CAN-19-0650 31744820

[164] Chan KK Oza AM Siu LL. The Statins as Anticancer Agents. Clin Cancer Res (2003) 9:10–9. 12538446

[165] Yang YF Jan YH Liu YP Yang CJ Su CY Chang YC. Squalene Synthase Induces Tumor Necrosis Factor Receptor 1 Enrichment in Lipid Rafts to Promote Lung Cancer Metastasis. Am J Respir Crit Care Med (2014) 190:675–87. 10.1164/rccm.201404-0714OC 25152164

[166] Parris TZ Kovács A Hajizadeh S Nemes S Semaan M Levin M. Frequent MYC Coamplification and DNA Hypomethylation of Multiple Genes on 8q in 8p11-p12-amplified Breast Carcinomas. Oncogenesis (2014) 3:e95. 10.1038/oncsis.2014.8 24662924 PMC4038389

[167] Liu Y Sun W Zhang K Zheng H Ma Y Lin D. Identification of Genes Differentially Expressed in Human Primary Lung Squamous Cell Carcinoma. Lung Cancer (2007) 56:307–17. 10.1016/j.lungcan.2007.01.016 17316888

[168] Yuen HF McCrudden CM Huang YH Tham JM Zhang X Zeng Q. TAZ Expression as a Prognostic Indicator in Colorectal Cancer. PloS One (2013) 8:e54211. 10.1371/journal.pone.0054211 23372686 PMC3553150

[169] Sui Z Zhou J Cheng Z Lu P. Squalene Epoxidase (SQLE) Promotes the Growth and Migration of the Hepatocellular Carcinoma Cells. Tumor Biol (2015) 36:6173–9. 10.1007/s13277-015-3301-x 25787749

[170] Brown DN Caffa I Cirmena G Piras D Garuti A Gallo M. Squalene Epoxidase is a Bona Fide Oncogene by Amplification With Clinical Relevance in Breast Cancer. Sci Rep (2016) 6:19435. 10.1038/srep19435 26777065 PMC4726025

[171] Garcia-Bermudez J Baudrier L Bayraktar EC Shen Y La K Guarecuco R. Squalene Accumulation in Cholesterol Auxotrophic Lymphomas Prevents Oxidative Cell Death. Nature (2019) 567:118–22. 10.1038/s41586-019-0945-5 PMC6405297 30760928

[172] Viswanathan VS Ryan MJ Dhruv HD Gill S Eichhoff OM Seashore-Ludlow B. Dependency of a Therapy-Resistant State of Cancer Cells on a Lipid Peroxidase Pathway. Nature (2017) 547:453–7. 10.1038/nature23007 PMC5667900 28678785

[173] Murai T. Cholesterol Lowering: Role in Cancer Prevention and Treatment. Biol Chem (2014) 396:1–11. 10.1515/hsz-2014-0194 25205720

[174] Xue T Zhang Y Zhang L Yao L Hu X Xu LX. Proteomic Analysis of Two Metabolic Proteins With Potential to Translocate to Plasma Membrane Associated With Tumor Metastasis Development and Drug Targets. J Proteome Res (2013) 12:1754–63. 10.1021/pr301100r 23445495

[175] Staedler D Chapuis-Bernasconi C Dehmlow H Fischer H Juillerat-Jeanneret L Aebi JD. Cytotoxic Effects of Combination of Oxidosqualene Cyclase Inhibitors With Atorvastatin in Human Cancer Cells. J Med Chem (2012) 55:4990–5002. 10.1021/jm300256z 22533316

[176] Mejia-Pous C Damiola F Gandrillon O. Cholesterol Synthesis-Related Enzyme Oxidosqualene Cyclase is Required to Maintain Self-Renewal in Primary Erythroid Progenitors. Cell Prolif (2011) 44:441–52. 10.1111/j.1365-2184.2011.00771.x PMC6495882 21951287

[177] Beckwitt CH Shiraha K Wells A. Lipophilic Statins Limit Cancer Cell Growth and Survival, Via Involvement of Akt Signaling. PloS One (2018) 13:e0197422. 10.1371/journal.pone.0197422 29763460 PMC5953490

[178] Hindler K Cleeland CS Rivera E Collard CD. The Role of Statins in Cancer Therapy. Oncologist (2006) 11:306–15. 10.1634/theoncologist.11-3-306 16549815

[179] Pisanti S Picardi P Ciaglia E D’Alessandro A Bifulco M. Novel Prospects of Statins as Therapeutic Agents in Cancer. Pharmacol Res (2014) 88:84–98. 10.1016/j.phrs.2014.06.013 25009097

[180] Gizzo S Quaranta M Nardelli GB Noventa M. Lipophilic Statins as Anticancer Agents: Molecular Targeted Actions and Proposal in Advanced Gynaecological Malignancies. Curr Drug Targets (2015) 16:1142–59. 10.2174/1389450116666150330113239 25901529

[181] Fatehi Hassanabad A. Current Perspectives on Statins as Potential Anti-Cancer Therapeutics: Clinical Outcomes and Underlying Molecular Mechanisms. Transl Lung Cancer Res (2019) 8:692–9. 10.21037/tlcr.2019.09.08 PMC6835101 31737505

[182] Osmak M. Statins and Cancer: Current and Future Prospects. Cancer Lett (2012) 324:1–12. 10.1016/j.canlet.2012.04.011 22542807

[183] AhmadiMAmiriSPecicSMachajFRosikJŁosMJ. Pleiotropic Effects of Statins: A Focus on Cancer. Biochim Biophys Acta BBA - Mol Basis Dis (2020) 1866:165968. 10.1016/j.bbadis.2020.165968 32927022

[184] Altwairgi AK. Statins Are Potential Anticancerous Agents (Review). Oncol Rep (2015) 33:1019–39. 10.3892/or.2015.3741 25607255

[185] Di Bello E Zwergel C Mai A Valente S. The Innovative Potential of Statins in Cancer: New Targets for New Therapies. Front Chem (2020) 8:516. 10.3389/fchem.2020.00516 32626692 PMC7312214

[186] Matusewicz L Meissner J Toporkiewicz M Sikorski AF. The Effect of Statins on Cancer Cells—Review. Tumor Biol (2015) 36:4889–904. 10.1007/s13277-015-3551-7 26002574

[187] Terzi H Altun A Şencan M. *In Vitro* Comparison of the Cytotoxic Effects of Statins on U266 Myeloma Cell Line. Indian J Med Res (2019) 150:630–4. 10.4103/ijmr.IJMR_672_18 PMC7038803 32048627

[188] Chen YH Chen YC Lin CC Hsieh YP Hsu CS Hsieh MC. Synergistic Anticancer Effects of Gemcitabine With Pitavastatin on Pancreatic Cancer Cell Line MIA PaCa-2 *In Vitro* and *In Vivo* . Cancer Manag Res (2020) 12:4645–65. 10.2147/CMAR.S247876 PMC7306478 32606957

[189] Lübtow MM Oerter S Quader S Jeanclos E Cubukova A Krafft M. *In Vitro* Blood–Brain Barrier Permeability and Cytotoxicity of an Atorvastatin-Loaded Nanoformulation Against Glioblastoma in 2D and 3D Models. Mol Pharm (2020) 17:1835–47. 10.1021/acs.molpharmaceut.9b01117 32315193

[190] Higgins MJ Prowell TM Blackford AL Byrne C Khouri NF Slater SA. A Short-Term Biomarker Modulation Study of Simvastatin in Women at Increased Risk of a New Breast Cancer. Breast Cancer Res Treat (2012) 131:915–24. 10.1007/s10549-011-1858-7 PMC3536477 22076478

[191] Kim ST Kang JH Lee J Park SH Park JO Park YS. Simvastatin Plus Capecitabine–Cisplatin Versus Placebo Plus Capecitabine–Cisplatin in Patients With Previously Untreated Advanced Gastric Cancer: A Double-Blind Randomised Phase 3 Study. Eur J Cancer (2014) 50:2822–30. 10.1016/j.ejca.2014.08.005 25218337

[192] Vinayak S Schwartz EJ Jensen K Lipson J Alli E McPherson L. A Clinical Trial of Lovastatin for Modification of Biomarkers Associated With Breast Cancer Risk. Breast Cancer Res Treat (2013) 142:389–98. 10.1007/s10549-013-2739-z PMC5508753 24166281

[193] Garwood ER Kumar AS Baehner FL Moore DH Au A Hylton N. Fluvastatin Reduces Proliferation and Increases Apoptosis in Women With High Grade Breast Cancer. Breast Cancer Res Treat (2010) 119:137–44. 10.1007/s10549-009-0507-x PMC4087110 19728082

[194] Beckwitt CH Brufsky A Oltvai ZN Wells A. Statin Drugs to Reduce Breast Cancer Recurrence and Mortality. Breast Cancer Res (2018) 20:144. 10.1186/s13058-018-1066-z 30458856 PMC6247616

[195] Farooqi MAM Malhotra N Mukherjee SD Sanger S Dhesy-Thind SK Ellis P. Statin Therapy in the Treatment of Active Cancer: A Systematic Review and Meta-Analysis of Randomized Controlled Trials. PloS One (2018) 13:e0209486. 10.1371/journal.pone.0209486 30571754 PMC6301687

[196] Brånvall E Ekberg S Eloranta S Wästerlid T Birmann BM Smedby KE. Statin Use is Associated With Improved Survival in Multiple Myeloma: A Swedish Population-Based Study of 4315 Patients. Am J Hematol (2020) 95:652–61. 10.1002/ajh.25778 32141627

[197] Murtola TJ Syvälä H Tolonen T Helminen M Riikonen J Koskimäki J. Atorvastatin Versus Placebo for Prostate Cancer Before Radical Prostatectomy—A Randomized, Double-blind, Placebo-Controlled Clinical Trial. Eur Urol (2018) 74:697–701. 10.1016/j.eururo.2018.06.037 30031572

[198] Bono AV Pagano F Montironi R Zattoni F Manganelli A Selvaggi FP. Effect of Complete Androgen Blockade on Pathologic Stage and Resection Margin Status of Prostate Cancer: Progress Pathology Report of the Italian PROSIT Study. Urology (2001) 57:117–21. 10.1016/S0090-4295(00)00866-9 11164155

[199] Matusewicz L Czogalla A Sikorski AF. Attempts to Use Statins in Cancer Therapy: An Update. Tumor Biol (2020) 42:101042832094176. 10.1177/1010428320941760 32662332

[200] Charlton-Menys V Durrington PN. Squalene Synthase Inhibitors: Clinical Pharmacology and Cholesterol-Lowering Potential. Drugs (2007) 67:11–6. 10.2165/00003495-200767010-00002 17209661

[201] Cenedella RJ Jacob R Borchman D Tang D Neely AR Samadi A. Direct Perturbation of Lens Membrane Structure may Contribute to Cataracts Caused by U18666A, an Oxidosqualene Cyclase Inhibitor. J Lipid Res (2004) 45:1232–41. 10.1194/jlr.M300469-JLR200 15102886

[202] Dey P Barros RPA Warner M Ström A Gustafsson J-Å. Insight Into the Mechanisms of Action of Estrogen Receptor β in the Breast, Prostate, Colon, and CNS. J Mol Endocrinol (2013) 51:T61–74. 10.1530/JME-13-0150101 24031087

[203] Dey P Ström A Gustafsson J-Å. Estrogen Receptor β Upregulates FOXO3a and Causes Induction of Apoptosis Through PUMA in Prostate Cancer. Oncogene (2014) 33:4213–25. 10.1038/onc.2013.384 24077289

[204] Singh V Sharma V Verma V Pandey D Yadav SK Maikhuri JP. Apigenin Manipulates the Ubiquitin–Proteasome System to Rescue Estrogen Receptor-β From Degradation and Induce Apoptosis in Prostate Cancer Cells. Eur J Nutr (2015) 54:1255–67. 10.1007/s00394-014-0803-z 25408199

[205] Pravettoni A Mornati O Martini PGV Marino M Colciago A Celotti F. Estrogen Receptor Beta (ERbeta) and Inhibition of Prostate Cancer Cell Proliferation: Studies on the Possible Mechanism of Action in DU145 Cells. Mol Cell Endocrinol (2007) 263:46–54. 10.1016/j.mce.2006.08.008 17023111

[206] Pandit J Danley DE Schulte GK Mazzalupo S Pauly TA Hayward CM. Crystal Structure of Human Squalene Synthase: A Key Enzyme in Cholesterol Biosynthesis. J Biol Chem (2000) 275:30610–7. 10.1074/jbc.M004132200 10896663

[207] Bergstrom JD Dufresne C Bills GF Nallin-Omstead M Byrne K. Discovery, Biosynthesis, and Mechanism of Action of the Zaragozic Acids: Potent Inhibitors of Squalene Synthase. Annu Rev Microbiol (1995) 49:607–39. 10.1146/annurev.mi.49.100195.003135 8561474

[208] He J Shin H Wei X Kadegowda AKG Chen R Xie SK. NPC1L1 Knockout Protects Against Colitis-Associated Tumorigenesis in Mice. BMC Cancer (2015) 15:189. 10.1186/s12885-015-1230-0 25881076 PMC4378275

[209] Huang J Li L Lian J Schauer S Vesely PW Kratky D. Tumor-Induced Hyperlipidemia Contributes to Tumor Growth. Cell Rep (2016) 15:336–48. 10.1016/j.celrep.2016.03.020 PMC4984953 27050512

[210] Gonias SL Karimi-Mostowfi N Murray SS Mantuano E Gilder AS. Expression of LDL Receptor-Related Proteins (LRPs) in Common Solid Malignancies Correlates With Patient Survival. PloS One (2017) 12:e0186649. 10.1371/journal.pone.0186649 29088295 PMC5663383

[211] Gallagher EJ Zelenko Z Neel BA Antoniou IM Rajan L Kase N. Elevated Tumor LDLR Expression Accelerates LDL Cholesterol-Mediated Breast Cancer Growth in Mouse Models of Hyperlipidemia. Oncogene (2017) 36:6462–71. 10.1038/onc.2017.247 PMC5690879 28759039

[212] Campion O Al Khalifa T Langlois B Thevenard-Devy J Salesse S Savary K. Contribution of the Low-Density Lipoprotein Receptor Family to Breast Cancer Progression. Front Oncol (2020) 10:882. 10.3389/fonc.2020.00882 32850302 PMC7406569

[213] Roslan Z Muhamad M Selvaratnam L Ab-Rahim S. The Roles of Low-Density Lipoprotein Receptor-Related Proteins 5, 6, and 8 in Cancer: A Review. J Oncol (2019) 2019:4536302. 10.1155/2019/4536302 31031810 PMC6457291

[214] Mooberry LK Sabnis NA Panchoo M Nagarajan B Lacko AG. Targeting the SR-B1 Receptor as a Gateway for Cancer Therapy and Imaging. Front Pharmacol (2016) 7:466. 10.3389/fphar.2016.00466 28018216 PMC5156841

[215] Feng H Wang M Wu C Yu J Wang D Ma J. High Scavenger Receptor Class B Type I Expression is Related to Tumor Aggressiveness and Poor Prognosis in Lung Adenocarcinoma: A STROBE Compliant Article. Medicine (Baltimore) (2018) 97:e0203. 10.1097/MD.0000000000010203 29595658 PMC5895397

[216] Schörghofer D Kinslechner K Preitschopf A Schütz B Röhrl C Hengstschläger M. The HDL Receptor SR-BI is Associated With Human Prostate Cancer Progression and Plays a Possible Role In Establishing Androgen Independence. Reprod Biol Endocrinol (2015) 13:88. 10.1186/s12958-015-0087-z 26251134 PMC4528807

[217] Wang C Li P Xuan J Zhu C Liu J Shan L. Cholesterol Enhances Colorectal Cancer Progression Via ROS Elevation and MAPK Signaling Pathway Activation. Cell Physiol Biochem (2017) 42:729–42. 10.1159/000477890 28618417

[218] Llaverias G Danilo C Mercier I Daumer K Capozza F Williams TM. Role of Cholesterol in the Development and Progression of Breast Cancer. Am J Pathol (2011) 178:402–12. 10.1016/j.ajpath.2010.11.005 PMC3069824 21224077

[219] Riscal R Skuli N Simon MC. Even Cancer Cells Watch Their Cholesterol! Mol Cell (2019) 76:220–31. 10.1016/j.molcel.2019.09.008 PMC7225778 31586545

[220] Kapourchali FR Surendiran G Goulet A Moghadasian MH. The Role of Dietary Cholesterol in Lipoprotein Metabolism and Related Metabolic Abnormalities: A Mini-Review. Crit Rev Food Sci Nutr (2016) 56:2408–15. 10.1080/10408398.2013.842887 26055276

[221] Püschel GP Henkel J. Dietary Cholesterol Does Not Break Your Heart But Kills Your Liver. Porto BioMed J (2018) 3:e12. 10.1016/j.pbj.0000000000000012 31595236 PMC6726297

[222] Quail DF Dannenberg AJ. The Obese Adipose Tissue Microenvironment in Cancer Development and Progression. Nat Rev Endocrinol (2019) 15:139–54. 10.1038/s41574-018-0126-x PMC6374176 30459447

[223] Cedó L Reddy ST Mato E Blanco-Vaca F Escolà-Gil JC. HDL and LDL: Potential New Players in Breast Cancer Development. J Clin Med (2019) 8:853. 10.3390/jcm8060853 PMC6616617 31208017

[224] Pelton K Coticchia CM Curatolo AS Schaffner CP Zurakowski D Solomon KR. Hypercholesterolemia Induces Angiogenesis and Accelerates Growth of Breast Tumors In Vivo. Am J Pathol (2014) 184:2099–110. 10.1016/j.ajpath.2014.03.006 PMC4076468 24952430

[225] Oh SH Choi SY Choi HJ Ryu HM Kim YJ Jung HY. The Emerging Role of Xanthine Oxidase Inhibition for Suppression of Breast Cancer Cell Migration and Metastasis Associated With Hypercholesterolemia. FASEB J (2019) 33:7301–14. 10.1096/fj.201802415RR 30860872

[226] Heilos D Röhrl C Pirker C Englinger B Baier D Mohr T. Altered Membrane Rigidity Via Enhanced Endogenous Cholesterol Synthesis Drives Cancer Cell Resistance to Destruxins. Oncotarget (2018) 9:25661–80. 10.18632/oncotarget.25432 PMC5986646 29876015

[227] Han T Lv Y Wang S Hu T Hong H Fu Z. PPARγ Overexpression Regulates Cholesterol Metabolism in Human L02 Hepatocytes. J Pharmacol Sci (2019) 139:1–8. 10.1016/j.jphs.2018.09.013 30554802

[228] Tachibana K Yamasaki D Ishimoto K Doi T. The Role of PPARs in Cancer. PPAR Res (2008) 2008:102737. 10.1155/2008/102737 18584037 PMC2435221

[229] Chinetti G Lestavel S Bocher V Remaley AT Neve B Torra IP. PPAR-α and PPAR-γ Activators Induce Cholesterol Removal From Human Macrophage Foam Cells Through Stimulation of the ABCA1 Pathway. Nat Med (2001) 7:53–8. 10.1038/83348 11135616

[230] Grabacka M Reiss K. Anticancer Properties of PPARα-Effects on Cellular Metabolism and Inflammation. PPAR Res (2008) 2008:930705. 10.1155/2008/930705 18509489 PMC2396219

[231] Gou Q Gong X Jin J Shi J Hou Y. Peroxisome Proliferator-Activated Receptors (PPARs) Are Potential Drug Targets for Cancer Therapy. Oncotarget (2017) 8:60704–9. 10.18632/oncotarget.19610 PMC5601172 28948004

[232] Zhao W Prijic S Urban BC Tisza MJ Zuo Y Li L. Candidate Antimetastasis Drugs Suppress the Metastatic Capacity of Breast Cancer Cells by Reducing Membrane Fluidity. Cancer Res (2016) 76:2037–49. 10.1158/0008-5472.CAN-15-1970 PMC8491548 26825169

[233] Preta G. New Insights Into Targeting Membrane Lipids for Cancer Therapy. Front Cell Dev Biol (2020) 8:571237. 10.3389/fcell.2020.571237 32984352 PMC7492565

[234] Zhang J Li Q Wu Y Wang D Xu L Zhang Y. Cholesterol Content in Cell Membrane Maintains Surface Levels of ErbB2 and Confers a Therapeutic Vulnerability in ErbB2-Positive Breast Cancer. Cell Commun Signal (2019) 17:15. 10.1186/s12964-019-0328-4 30786890 PMC6383291

[235] Aguirre-Portolés C Feliu J Reglero G Ramírez de Molina A. ABCA1 Overexpression Worsens Colorectal Cancer Prognosis by Facilitating Tumour Growth and Caveolin-1-Dependent Invasiveness, and These Effects can be Ameliorated Using the BET Inhibitor Apabetalone. Mol Oncol (2018) 12:1735–52. 10.1002/1878-0261.12367 PMC6166002 30098223

[236] Koundouros N Poulogiannis G. Reprogramming of Fatty Acid Metabolism in Cancer. Br J Cancer (2020) 122:4–22. 10.1038/s41416-019-0650-z 31819192 PMC6964678

[237] Adam RM Mukhopadhyay NK Kim J Di Vizio D Cinar B Boucher K. Cholesterol Sensitivity of Endogenous and Myristoylated Akt. Cancer Res (2007) 67:6238–46. 10.1158/0008-5472.CAN-07-0288 17616681

[238] Chen L Peng J Wang Y Jiang H Wang W Dai J. Fenofibrate-Induced Mitochondrial Dysfunction and Metabolic Reprogramming Reversal: The Anti-Tumor Effects in Gastric Carcinoma Cells Mediated by the PPAR Pathway. Am J Transl Res (2020) 12:428–46. PMC7061836 32194894

[239] Ertunc ME Hotamisligil GS. Lipid Signaling and Lipotoxicity in Metaflammation: Indications for Metabolic Disease Pathogenesis and Treatment. J Lipid Res (2016) 57:2099–114. 10.1194/jlr.R066514 PMC5321214 27330055

[240] Wang YJ Bian Y Luo J Lu M Xiong Y Guo SY. Cholesterol and Fatty Acids Regulate Cysteine Ubiquitylation of ACAT2 Through Competitive Oxidation. Nat Cell Biol (2017) 19:808–19. 10.1038/ncb3551 PMC5518634 28604676

[241] Jarc E Petan T. Lipid Droplets and the Management of Cellular Stress. Yale J Biol Med (2019) 92:435–52. PMC6747940 31543707

[242] Qiu B Ackerman D Sanchez DJ Li B Ochocki JD Grazioli A. HIF2α-Dependent Lipid Storage Promotes Endoplasmic Reticulum Homeostasis in Clear-Cell Renal Cell Carcinoma. Cancer Discov (2015) 5:652–67. 10.1158/2159-8290.CD-14-1507 PMC4456212 25829424

[243] Yue S Li J Lee SY Lee HJ Shao T Song B. Cholesteryl Ester Accumulation Induced by PTEN Loss and PI3K/AKT Activation Underlies Human Prostate Cancer Aggressiveness. Cell Metab (2014) 19:393–406. 10.1016/j.cmet.2014.01.019 24606897 PMC3969850

[244] de Gonzalo-Calvo D López-Vilaró L Nasarre L Perez-Olabarria M Vázquez T Escuin D. Intratumor Cholesteryl Ester Accumulation is Associated With Human Breast Cancer Proliferation and Aggressive Potential: A Molecular and Clinicopathological Study. BMC Cancer (2015) 15:460. 10.1186/s12885-015-1469-5 26055977 PMC4460760

[245] Mulas MF Abete C Pulisci D Pani A Massidda B Dessì S. Cholesterol Esters as Growth Regulators of Lymphocytic Leukaemia Cells. Cell Prolif (2011) 44:360–71. 10.1111/j.1365-2184.2011.00758.x PMC6496738 21645151

[246] Bemlih S Poirier M-D El Andaloussi A. Acyl-Coenzyme A: Cholesterol Acyltransferase Inhibitor Avasimibe Affect Survival and Proliferation of Glioma Tumor Cell Lines. Cancer Biol Ther (2010) 9:1025–32. 10.4161/cbt.9.12.11875 20404512

[247] Tirinato L Liberale C Di Franco S Candeloro P Benfante A La Rocca R. Lipid Droplets: A New Player in Colorectal Cancer Stem Cells Unveiled by Spectroscopic Imaging. Stem Cells (2015) 33:35–44. 10.1002/stem.1837 25186497 PMC4311668

[248] Jiang Y Sun A Zhao Y Ying W Sun H Yang X. Proteomics Identifies New Therapeutic Targets of Early-Stage Hepatocellular Carcinoma. Nature (2019) 567:257–61. 10.1038/s41586-019-0987-8 30814741

[249] Saraon P Cretu D Musrap N Karagiannis GS Batruch I Drabovich AP. Quantitative Proteomics Reveals That Enzymes of the Ketogenic Pathway are Associated With Prostate Cancer Progression. Mol Cell Proteomics (2013) 12:1589–601. 10.1074/mcp.M112.023887 PMC3675816 23443136

[250] Lacombe AMF Soares IC Mariani BM de P Nishi MY Bezerra-Neto JE Charchar H da S. Sterol O-Acyl Transferase 1 as a Prognostic Marker of Adrenocortical Carcinoma. Cancers (2020) 12:247. 10.3390/cancers12010247 PMC7016635 31963898

[251] Warner GJ Stoudt G Bamberger M Johnson WJ Rothblat GH. Cell Toxicity Induced by Inhibition of Acyl Coenzyme A:Cholesterol Acyltransferase and Accumulation of Unesterified Cholesterol. J Biol Chem (1995) 270:5772–8. 10.1074/jbc.270.11.5772 7890706

[252] Wang J Tan M Ge J Zhang P Zhong J Tao L. Lysosomal Acid Lipase Promotes Cholesterol Ester Metabolism and Drives Clear Cell Renal Cell Carcinoma Progression. Cell Prolif (2018) 51:e12452. 10.1111/cpr.12452 29569766 PMC6528899

[253] Chen Y Hughes-Fulford M. Human Prostate Cancer Cells Lack Feedback Regulation of Low-Density Lipoprotein Receptor and its Regulator, SREBP2. Int J Cancer (2001) 91:41–5. 10.1002/1097-0215(20010101)91:1<41::AID-IJC1009>3.0.CO;2-2 11149418

[254] Locke JA Guns ES Lubik AA Adomat HH Hendy SC Wood CA. Androgen Levels Increase by Intratumoral De Novo Steroidogenesis During Progression of Castration-Resistant Prostate Cancer. Cancer Res (2008) 68:6407–15. 10.1158/0008-5472.CAN-07-5997 18676866

[255] Leon CG Locke JA Adomat HH Etinger SL Twiddy AL Neumann RD. Alterations in Cholesterol Regulation Contribute to the Production of Intratumoral Androgens During Progression to Castration-Resistant Prostate Cancer in a Mouse Xenograft Model. Prostate (2010) 70:390–400. 10.1002/pros.21072 19866465

[256] Martinez-Outschoorn UE Lin Z Whitaker-Menezes D Howell A Sotgia F Lisanti MP. Ketone Body Utilization Drives Tumor Growth and Metastasis. Cell Cycle (2012) 11:3964–71. 10.4161/cc.22137 PMC3507492 23082722

[257] Ozsvari B Sotgia F Simmons K Trowbridge R Foster R Lisanti MP. Mitoketoscins: Novel Mitochondrial Inhibitors for Targeting Ketone Metabolism in Cancer Stem Cells (CSCs). Oncotarget (2017) 8:78340–50. 10.18632/oncotarget.21259 PMC5667966 29108233

[258] Lo YW Lin ST Chang SJ Chan CH Lyu KW Chang JF. Mitochondrial Proteomics With siRNA Knockdown to Reveal ACAT1 and MDH2 in the Development of Doxorubicin-Resistant Uterine Cancer. J Cell Mol Med (2015) 19:744–59. 10.1111/jcmm.12388 PMC4395189 25639359

[259] Dong Y Tu R Liu H Qing G. Regulation of Cancer Cell Metabolism: Oncogenic MYC in the Driver’s Seat. Signal Transduct Target Ther (2020) 5:124. 10.1038/s41392-020-00235-2 32651356 PMC7351732

[260] Zhong C Fan L Yao F Shi J Fang W Zhao H. HMGCR is Necessary for the Tumorigenecity of Esophageal Squamous Cell Carcinoma and Is Regulated by Myc. Tumor Biol (2014) 35:4123–9. 10.1007/s13277-013-1539-8 24390662

[261] Cheng C Geng F Cheng X Guo D. Lipid Metabolism Reprogramming and its Potential Targets in Cancer. Cancer Commun Lond Engl (2018) 38:27. 10.1186/s40880-018-0301-4 PMC5993136 29784041

[262] Haskins JW Zhang S Means RE Kelleher JK Cline GW Canfrán-Duque A. Neuregulin-Activated ERBB4 Induces the SREBP-2 Cholesterol Biosynthetic Pathway and Increases Low-Density Lipoprotein Uptake. Sci Signal (2015) 8:ra111. 10.1126/scisignal.aac5124 26535009 PMC4666504

[263] Bakiri L Hamacher R Graña O Guío-Carrión A Campos-Olivas R Martinez L. Liver Carcinogenesis by FOS-Dependent Inflammation and Cholesterol Dysregulation. J Exp Med (2017) 214:1387–409. 10.1084/jem.20160935 PMC5413325 28356389

[264] Kloudova A Guengerich PF Soucek P. The Role of Oxysterols in Human Cancer. Trends Endocrinol Metab (2017) 28:485–96. 10.1016/j.tem.2017.03.002 PMC5474130 28410994

[265] Olkkonen VM Béaslas O Nissilä E. Oxysterols and Their Cellular Effectors. Biomolecules (2012) 2:76–103. 10.3390/biom2010076 24970128 PMC4030866

[266] He S Nelson ER. 27-Hydroxycholesterol, an Endogenous Selective Estrogen Receptor Modulator. Maturitas (2017) 104:29–35. 10.1016/j.maturitas.2017.07.014 28923174 PMC5657610

[267] Raza S Ohm JE Dhasarathy A Schommer J Roche C Hammer KDP. The Cholesterol Metabolite 27-Hydroxycholesterol Regulates p53 Activity and Increases Cell Proliferation Via MDM2 in Breast Cancer Cells. Mol Cell Biochem (2015) 410:187–95. 10.1007/s11010-015-2551-7 PMC4821067 26350565

[268] Shen Z Zhu D Liu J Chen J Liu Y Hu C. 27-Hydroxycholesterol Induces Invasion and Migration of Breast Cancer Cells by Increasing MMP9 and Generating EMT Through Activation of STAT-3. Environ Toxicol Pharmacol (2017) 51:1–8. 10.1016/j.etap.2017.02.001 28257824

[269] Zhu D Shen Z Liu J Chen J Liu Y Hu C. The ROS-mediated Activation of STAT-3/VEGF Signaling is Involved in the 27-Hydroxycholesterol-Induced Angiogenesis in Human Breast Cancer Cells. Toxicol Lett (2016) 264:79–86. 10.1016/j.toxlet.2016.11.006 27856279

[270] Revilla G Pons M de P Baila-Rueda L García-León A Santos D Cenarro A. Cholesterol and 27-Hydroxycholesterol Promote Thyroid Carcinoma Aggressiveness. Sci Rep (2019) 9:10260. 10.1038/s41598-019-46727-2 31311983 PMC6635382

[271] Clendening JW Pandyra A Boutros PC El Ghamrasni S Khosravi F Trentin GA. Dysregulation of the Mevalonate Pathway Promotes Transformation. Proc Natl Acad Sci (2010) 107:15051–6. 10.1073/pnas.0910258107 PMC2930553 20696928

[272] Xue L Qi H Zhang H Ding L Huang Q Zhao D. Targeting SREBP-2-Regulated Mevalonate Metabolism for Cancer Therapy. Front Oncol (2020) 10:1510. 10.3389/fonc.2020.01510 32974183 PMC7472741

[273] Zhong C Fan L Li Z Yao F Zhao H. SREBP2 is Upregulated in Esophageal Squamous Cell Carcinoma and Co−Operates With C−Myc to Regulate HMGCR Expression. Mol Med Rep (2019) 20:3003–10. 10.3892/mmr.2019.10577 PMC6755167 31432128

[274] Lewis CA Brault C Peck B Bensaad K Griffiths B Mitter R. SREBP Maintains Lipid Biosynthesis and Viability of Cancer Cells Under Lipid- and Oxygen-Deprived Conditions and Defines a Gene Signature Associated With Poor Survival in Glioblastoma Multiforme. Oncogene (2015) 34:5128–40. 10.1038/onc.2014.439 25619842

[275] Porstmann T Santos CR Griffiths B Cully M Wu M Leevers S. SREBP Activity is Regulated by mTORC1 and Contributes to Akt-Dependent Cell Growth. Cell Metab (2008) 8:224–36. 10.1016/j.cmet.2008.07.007 PMC2593919 18762023

[276] Ricoult SJH Yecies JL Ben-Sahra I Manning BD. Oncogenic PI3K and K-Ras Stimulate De Novo Lipid Synthesis Through mTORC1 and SREBP. Oncogene (2016) 35:1250–60. 10.1038/onc.2015.179 PMC4666838 26028026

[277] Dong F Mo Z Eid W Courtney KC Zha X. Akt Inhibition Promotes ABCA1-Mediated Cholesterol Efflux to ApoA-I Through Suppressing mTORC1. PloS One (2014) 9:e113789. 10.1371/journal.pone.0113789 25415591 PMC4240609

[278] Moon SH Huang CH Houlihan SL Regunath K Freed-Pastor WA Morris JP 4th. P53 Represses the Mevalonate Pathway to Mediate Tumor Suppression. Cell (2019) 176:564–80.e19. 10.1016/j.cell.2018.11.011 30580964 PMC6483089

[279] Peck B Schulze A. Lipid Metabolism at the Nexus of Diet and Tumor Microenvironment. Trends Cancer (2019) 5:693–703. 10.1016/j.trecan.2019.09.007 31735288

[280] Ingallina E Sorrentino G Bertolio R Lisek K Zannini A Azzolin L. Mechanical Cues Control Mutant p53 Stability Through a Mevalonate–RhoA Axis. Nat Cell Biol (2018) 20:28–35. 10.1038/s41556-017-0009-8 29255172 PMC6179142

[281] Zhuang L Kim J Adam RM Solomon KR Freeman MR. Cholesterol Targeting Alters Lipid Raft Composition and Cell Survival in Prostate Cancer Cells and Xenografts. J Clin Invest (2005) 115:959–68. 10.1172/JCI200519935 PMC1064980 15776112

[282] Jia Y Wang Y Xie J. The Hedgehog Pathway: Role in Cell Differentiation, Polarity and Proliferation. Arch Toxicol (2015) 89:179–91. 10.1007/s00204-014-1433-1 PMC4630008 25559776

[283] Guo D Reinitz F Youssef M Hong C Nathanson D Akhavan D. An LXR Agonist Promotes Glioblastoma Cell Death Through Inhibition of an EGFR/AKT/SREBP-1/LDLR-Dependent Pathway. Cancer Discov (2011) 1:442–56. 10.1158/2159-8290.CD-11-0102 PMC3207317 22059152

[284] Gholkar AA Cheung K Williams KJ Lo YC Hamideh SA Nnebe C. Fatostatin Inhibits Cancer Cell Proliferation by Affecting Mitotic Microtubule Spindle Assembly and Cell Division. J Biol Chem (2016) 291:17001–8. 10.1074/jbc.C116.737346 PMC5016105 27378817

[285] Wen D Wang J Van Den Driessche G Chen Q Zhang Y Chen G. Adipocytes as Anticancer Drug Delivery Depot. Matter (2019) 1:1203–14. 10.1016/j.matt.2019.08.007

[286] Shen H Shi S Zhang Z Gong T Sun X. Coating Solid Lipid Nanoparticles With Hyaluronic Acid Enhances Antitumor Activity Against Melanoma Stem-Like Cells. Theranostics (2015) 5:755–71. 10.7150/thno.10804 PMC4402499 25897340

[287] Sobot D Mura S Rouquette M Vukosavljevic B Cayre F Buchy E. Circulating Lipoproteins: A Trojan Horse Guiding Squalenoylated Drugs to LDL-Accumulating Cancer Cells. Mol Ther (2017) 25:1596–605. 10.1016/j.ymthe.2017.05.016 PMC5498828 28606375

[288] Mooberry LK Nair M Paranjape S McConathy WJ Lacko AG. Receptor Mediated Uptake of Paclitaxel From a Synthetic High Density Lipoprotein Nanocarrier. J Drug Target (2010) 18:53–8. 10.3109/10611860903156419 19637935

[289] Radwan AA Alanazi FK. Targeting Cancer Using Cholesterol Conjugates. Saudi Pharm J (2014) 22:3–16. 10.1016/j.jsps.2013.01.003 24493968 PMC3909757

